# Altered Brain Network Dynamics in Schizophrenia Patients With Predominant Negative Symptoms: A Resting‐State fMRI Study Using Co‐Activation Pattern Analysis

**DOI:** 10.1002/hbm.70369

**Published:** 2025-09-24

**Authors:** Xingsong Wang, Yao Zhang, Pei‐juan Wang, Qi Yan, Xiao‐xiao Wang, Hai‐su Wu, Shuai‐biao Li, Min‐yi Chu, Yi Wang, Simon S. Y. Lui, Qin‐yu Lv, Li Kong, Zheng‐hui Yi, Raymond C. K. Chan

**Affiliations:** ^1^ School of Psychology Shanghai Normal University Shanghai China; ^2^ Shanghai Mental Health Centre Shanghai Jiao Tong University School of Medicine Shanghai China; ^3^ Department of Psychiatry Huashan Hospital, Fudan University Shanghai China; ^4^ Nantong Fourth People's Hospital Nantong China; ^5^ Neuropsychology and Applied Cognitive Neuroscience Laboratory, CAS Key Laboratory of Mental Health, Institute of Psychology Chinese Academy of Sciences Beijing China; ^6^ Department of Psychology University of Chinese Academy of Sciences Beijing China; ^7^ Department of Psychiatry, School of Clinical Medicine The University of Hong Kong Hong Kong Special Administrative Region China; ^8^ Institute of Mental Health Fudan University Shanghai China

**Keywords:** brain networks, co‐activation pattern analysis, negative symptoms, network dynamics, resting‐state fMRI, schizophrenia

## Abstract

Negative symptoms remain a major therapeutic challenge in schizophrenia, significantly impacting functional outcomes, yet their underlying neural mechanisms remain poorly understood. Traditional static functional connectivity analyses, which examine average correlations over time, may overlook critical temporal features of brain network organization and fail to capture dynamic shifts in connectivity patterns. Resting‐state functional magnetic resonance imaging (rs‐fMRI), particularly when analyzed using co‐activation pattern analysis (CAP), provides a framework to study these dynamic network changes with greater temporal resolution. Using CAP analysis of rs‐fMRI data, we investigated brain network dynamics in 31 schizophrenia patients with predominant negative symptoms, 31 patients without predominant negative symptoms, and 34 healthy controls. Eight distinct brain states were identified, characterized by antagonistic relationships between sensorimotor, default mode, and salience networks. Compared to healthy controls, the overall schizophrenia group showed altered temporal characteristics, including a reduced occurrence of a sensorimotor‐dominant state and excessive transitions from this state to a control‐salience network state. Notably, patients with predominant negative symptoms demonstrated distinct temporal characteristics, including reduced dwell time in sensorimotor‐salience states and excessive transitions from sensorimotor to control‐salience network states. In contrast, patients without predominant negative symptoms did not exhibit such excessive state transitions, while their symptom severity correlated with the occurrence of a cognitive‐sensorimotor network state. Network alterations significantly correlated with symptom severity in both the overall schizophrenia group and the subgroup without predominant negative symptoms, while no significant correlations were observed in patients with predominant negative symptoms. These findings suggest that predominant negative symptoms are associated with stable trait‐like network reorganization characterized by excessive state transitions rather than state‐dependent dysregulation, providing potential neuroimaging markers for clinical assessment.

## Graphical abstract

Co‐activation pattern analysis reveals schizophrenia patients with predominant negative symptoms exhibit distinct brain dynamics: impaired sensorimotor–visual stability, reduced sensorimotor–salience maintenance, and excessive sensorimotor‐to‐control transitions. These alterations, independent of symptom severity, represent stable trait‐like neurobiological markers rather than state‐dependent dysregulation, offering potential neuroimaging biomarkers for assessment.

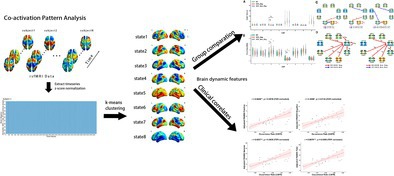

**Summary**

- Network Dynamics in Negative Symptoms: Patients with predominant negative symptoms demonstrated a unique neural signature, characterized by both spatial and temporal alterations, including reduced stability of a sensorimotor–visual state and excessive transitions from sensorimotor to control–salience network states, suggesting a unique neural signature for negative symptomatology.
- State‐Dependent versus Trait‐Like Changes: While the network dynamics of patients without predominant negative symptoms correlate with symptom severity, suggesting a state‐dependent dysregulation, those with predominant negative symptoms exhibit stable trait‐like network reorganization characterized by excessive state transitions, indicating different underlying mechanisms.
- Clinical‐Neural Correlations: Network alterations significantly correlate with symptom severity in both the overall schizophrenia group and the subgroup without predominant negative symptoms, while no significant correlations were observed in patients with predominant negative symptoms, suggesting potential utility as neuroimaging markers for clinical assessment.

## 1 Introduction

Negative symptoms remain one of the most challenging aspects of schizophrenia to address, despite significant advancements in psychopharmacology. Affecting approximately 50%–60% of individuals with schizophrenia, these symptoms significantly impact clinical and functional outcomes, including long‐term quality of life (Chan et al. 2022; Galderisi et al. 2018). Negative symptoms, characterized by diminished emotional expression and motivational deficits (Correll and Schooler 2020; Marder and Galderisi 2017; Strauss et al. 2019), are often resistant to current therapeutic approaches, unlike positive symptoms that typically respond to antipsychotics (Fusar‐Poli et al. 2014; Remington et al. 2016). This treatment resistance, combined with the complexity and heterogeneity of symptom presentation, underscores the urgent need for objective biomarkers to better characterize and manage negative symptoms (Krause et al. 2018; Millan et al. 2014). Such biomarkers could guide the development of personalized treatment strategies, particularly for individuals with predominant negative symptoms (Galderisi et al. 2015; Remington et al. 2016).

Resting‐state functional magnetic resonance imaging (rs‐fMRI) has provided crucial insights into altered brain connectivity in schizophrenia, with consistent findings of dysconnectivity in networks such as the default mode network (DMN), ventral attention network (VAN), and frontoparietal networks (FPN) (Dong et al. 2017; Li et al. 2019; Woodward et al. 2011). While static functional connectivity linked specific network alterations to negative symptom severity—particularly in reward‐related regions (ventral striatum and orbitofrontal cortex) and DMN (Giordano et al. 2023; Wang, Chang, and Wang 2023; Wang, Zhang, et al. 2023), these approaches assume temporal stationarity, potentially overlooking the dynamic nature of brain network interactions (Hutchison et al. 2013; Lurie et al. 2020; Preti et al. 2017). Motivation and expressive deficits have been suggested to arise from different neural correlates within these networks (Giuliani et al. 2024; Saleh et al. 2021). In contrast, dynamic connectivity analyses have demonstrated the temporal variability of network configurations (Di and Biswal 2015; Hutchison et al. 2013), revealing critical insights into schizophrenia‐related dysconnectivity (Du et al. 2018; Yu et al. 2015). While sliding‐window approaches have been widely adopted in dynamic connectivity studies, they face inherent limitations in temporal resolution and stability, particularly when characterizing rapid state transitions (Keilholz et al. 2017; Leonardi and Van De Ville 2015; Lurie et al. 2020). Moreover, existing dynamic methods vary in their ability to balance temporal sensitivity with signal‐to‐noise considerations (Hindriks et al. 2016; Shakil et al. 2016), which may partly explain the limited application of these methods in studying negative symptoms. These methodological challenges highlight the need for advanced analytical approaches that can reliably capture both temporal dynamics and spatial patterns of brain network organization (Lurie et al. 2020; Preti et al. 2017).

Co‐activation pattern (CAP) analysis offers a promising alternative by combining frame‐wise temporal resolution with the ability to preserve whole‐brain spatial patterns (Cohen et al. 2021; Kaiser et al. 2019; Liu et al. 2018). Originally developed from point process analysis approaches (Liu and Duyn 2013; Tagliazucchi et al. 2012), CAP analysis conceptualizes brain dynamics through distinct snapshots of neural activity, where individual fMRI volumes are clustered based on their spatial similarity to identify recurring activation patterns. Unlike traditional static methods or sliding window approaches that average connectivity across arbitrary time windows, CAP analysis preserves the native temporal resolution of fMRI data by treating each volume as an independent sample of brain activity. This data‐driven method overcomes key limitations of traditional sliding‐window approaches by directly capturing instantaneous brain states without temporal smoothing artifacts, enabling robust detection of recurring network configurations and their temporal evolution (Kaiser et al. 2019; Murray et al. 2021). Technically, the CAP methodology involves normalizing time series data, applying clustering algorithms (typically k‐means) to identify representative brain states, and assigning each time frame to its best‐matching state based on spatial correlation (Liu et al. 2018). Through this clustering process, the method identifies representative activation patterns or states and characterizes their spatial configuration and temporal sequence, allowing researchers to track how the brain transitions between different functional configurations over time. CAP analysis has demonstrated unique strengths in quantifying rapid state transitions through multiple dynamic metrics, including dwell time, occurrence rate, and transition probabilities (An et al. 2024; Sun et al. 2024). The high temporal sensitivity of this method allows detection of subtle variations in network engagement patterns while maintaining spatial specificity across distributed brain systems (Zhang et al. 2024). These attributes position CAP analysis as a powerful tool for identifying disease‐specific alterations in brain network dynamics and their associations with clinical symptoms (Janes et al. 2020; Mentink et al. 2021; Yang, Tang, et al. 2021; Yang, Zhang, et al. 2021).

This study aimed to investigate the dynamic network characteristics associated with predominant negative symptoms in schizophrenia using CAP analysis. Specifically, we explored spatial stability, temporal dynamics, and state transition patterns to uncover potential neural mechanisms underlying negative symptomatology. Furthermore, we sought to evaluate the utility of dynamic network features as candidate neuroimaging biomarkers for assessing symptom severity and guiding targeted interventions. Based on the evidence of altered temporal dynamics in schizophrenia, we hypothesize that patients with predominant negative symptoms will exhibit distinct CAP temporal profiles, including reduced stability and altered transitions among key networks such as the DMN, salience network, and sensorimotor network, compared to both patients without predominant negative symptoms and healthy controls.

## 2 Materials and Methods

### 2.1 Participants and Clinical Assessment

Patients were recruited from the Shanghai Mental Health Centre, and diagnoses of schizophrenia were confirmed using the Structured Clinical Interview for DSM‐5 (Regier et al. 2013). After excluding one schizophrenia patient with excessive head motion (maximum translation or rotation greater than 2.5 mm or 2.5°), the final sample included 96 participants: 34 healthy controls (HC; 21 males, mean age 26.1 ± 3.2 years, mean education 14.9 ± 3.7 years), 31 schizophrenia patients with predominantly negative symptoms (SCH_Neg; 21 males, mean age 24.6 ± 5.2 years, mean education 12.4 ± 2.1 years), and 31 schizophrenia patients with non‐predominant negative symptoms (SCH_Non_Neg; 22 males, mean age 25.0 ± 5.6 years, mean education 13.2 ± 2.7 years).

All participants were right‐handed, assessed using the Edinburgh Handedness Inventory (Oldfield 1971). Clinical symptoms were assessed using the Positive and Negative Syndrome Scale (PANSS) (Kay et al. 1987). The SCH_Neg group was defined by a PANSS negative symptoms subscale score of > 3 on at least 3 items or > 4 on at least 2 items, with a PANSS positive symptom subscale score of < 19 (Kong et al. 2024; Rabinowitz et al. 2013; Stauffer et al. 2012). The SCH_Non_Neg group consisted of patients not meeting the criteria for predominant negative symptoms. The HC group was recruited from the local community and screened to exclude individuals with personal histories of psychiatric disorders, neurological diseases, significant head injuries, or recent substance abuse. All participants provided written informed consent, and the study was approved by the Ethics Committee of Shanghai Mental Health Centre approved the research protocol (No. 2021‐50).

### 2.2 MRI Data Acquisition

Neuroimaging data were collected using a 3‐Tesla MR scanner (Verio, Erlangen, Germany) at the Shanghai Mental Health Centre. High‐resolution T1‐weighted structural images were acquired through a magnetization‐prepared rapid gradient‐echo (MPRAGE) sequence with the following parameters: repetition time (TR) = 2530 ms, echo time (TE) = 1.66 ms, flip angle = 7°, field of view (FOV) = 256 × 256 mm, image matrix = 256 × 256, and voxel size = 1 × 1 × 1 mm^3^, and 182 sagittal slices (total scan time ≈ 340 s). Functional MRI data were obtained using a planar echo imaging oxygen saturation dependence (ep2D bold) sequence with TR = 2600 ms, TE = 30 ms, acquisition matrix = 64 × 64, flip angle = 90°, slice thickness = 3.5 mm, FOV = 448 × 448 mm, voxel size = 3.1 × 3.1 × 3.5 mm, and 40 slices, and 180 volumes (total scan time = 468 s). During the resting‐state fMRI scan, all participants were instructed to keep their eyes closed, stay awake, and avoid thinking about anything specific.

### 2.3 fMRI Data Preprocessing

fMRI data preprocessing was performed using DPARSF (http://rfmri.org/DPARSF) (Yan and Zang 2010). The preprocessing steps included the following: (1) removal of the first 10 time points; (2) slice timing correction and realignment; (3) co‐registration of T1 images to fMRI data; (4) segmentation of T1 images into grey matter, white matter, and cerebrospinal fluid; (5) spatial normalization of the fMRI data to MNI space; (6) nuisance regression using 24 head motion parameters, mean white matter (WM), and mean cerebrospinal fluid (CSF) signal; (7) detrending; (8) band‐pass filter (0.01–0.08 Hz); and (9) spatial smoothing with a 6 mm FWHM Gaussian kernel.

Following preprocessing, region‐specific time series were extracted from the preprocessed functional data by averaging the BOLD signals across all voxels within each of the 408 regions of interest (ROIs). The parcellation scheme integrated 400 cortical regions from the Schaefer atlas (Schaefer et al. 2018), which is based on Yeo's 7‐network parcellation (Thomas Yeo et al. 2011), along with 8 subcortical regions (bilateral caudate nucleus, putamen, globus pallidus, and amygdala) from the Automated Anatomical Labeling (AAL) atlas (Tzourio‐Mazoyer et al. 2002). These 8 subcortical regions were treated as a separate network, referred to as the subcortical network (SCN). This comprehensive 408‐region parcellation was selected to provide a balanced representation of cortical and subcortical structures while maintaining comparable spatial resolution across regions (Yang, Tang, et al. 2021; Yang, Zhang, et al. 2021). The cortical parcellation encompasses seven canonical networks: visual (VN), somatomotor (SMN), dorsal attention (DAN), salience/ventral attention (SAN), limbic (LN), control (CN), and default mode (DMN) networks. These networks were originally derived using a clustering algorithm on resting‐state functional connectivity data (Thomas Yeo et al. 2011).

### 2.4 Co‐Activation Pattern (CAP) Analysis

CAP analysis was implemented as a frame‐wise analytical approach that treats each fMRI volume as an independent spatial configuration, enabling direct characterization of momentary brain states without temporal averaging. In this framework, a CAP center represents a characteristic brain activation pattern identified through clustering, while a CAP state indicates the assignment of individual time points to their best‐matching pattern. The CAP analysis was conducted to investigate dynamic brain functional connectivity using a region of interest (ROI) approach, employing the open‐source “capcalc” package (Frederick, B, capcalc [Computer Software] (2016‐2022). Available from https://github.com/bbfrederick/capcalc). This package, referenced in Janes et al. (2020), is based on earlier versions of the CAP pipeline reported in Kaiser et al. (2019). The analysis involved several key steps: (1) *Z* score normalization was applied to individual subject time series to eliminate scale differences between ROIs; (2) All time series from HC group subjects were concatenated into a large‐scale matrix; (3) An improved k‐means clustering algorithm was applied to identify co‐activation patterns (CAPs), treating each time point as a 408‐dimensional vector; (4) The optimal number of clusters was determined by testing a range from 2 to 20 clusters and evaluating multiple metrics, including the silhouette coefficient (Rousseeuw 1987), Calinski‐Harabasz index (Caliński and Harabasz 1974), Davies‐Bouldin index (Davies and Bouldin 1979), sum of squared errors, average state transition probability, and mean error (see Supporting Information for further details); (5) Clustering was implemented with 100 iterations, 5 random initializations, and a distance measure based on 1 minus Pearson's correlation coefficient to capture similarity in spatial activation patterns; (6) Dimensionality reduction was conducted through principal component analysis (PCA) prior to clustering, using the default settings which retain 8 principal components, balancing data reduction with information preservation; (7) The resulting cluster centers were mapped back to brain space for visualization and interpretation, with normalized *Z* value maps ranging from −1 to 1. To ensure consistent state definitions across groups while quantifying potential alterations in patient populations, the clustering pattern derived from the HC group was subsequently applied to the schizophrenia groups by calculating the spatial similarity for each frame between schizophrenia groups and the normalized CAP patterns derived from the HC group (Yang, Tang, et al. 2021; Yang, Zhang, et al. 2021). Each frame was assigned to the CAP state with the highest similarity using Pearson's correlation.

### 2.5 Spatial Stability of CAP States

The spatial stability analysis quantifies the consistency of activation patterns within each CAP state, providing insights into the reliability of network configurations across time and subjects (see Supporting Information for detailed calculations). The spatial stability of each CAP state was assessed using two complementary methods. First, the individual stable activation rate (iSAR) was calculated for stable regions within each CAP state. Stable regions were identified at the group level as areas showing consistent positive (threshold > 0.4) or negative (threshold < −0.4) activation in more than 50% of subjects. For each subject, iSAR was then calculated as the proportion of frames maintaining stable positive or negative activation in these regions (Zhang et al. 2024). Second, global stability was evaluated by calculating the distance to the CAP center for each frame (i.e., each time point of whole‐brain activity), defined as 1—*r*, where *r* is the Pearson correlation between the frame and the CAP center. A lower mean distance indicates higher overall spatial stability. Together, these metrics offer a comprehensive view of CAP state stability, addressing both regional and global consistency.

### 2.6 Temporal Dynamics of CAP States

The temporal dynamics analysis characterizes how brain states evolve over time, quantifying both the persistence of individual states and the patterns of transitions between different network configurations (see Supporting Information for methodological details). To investigate the temporal characteristics of CAP states, several dynamic features were extracted at the individual level. The dwell time, defined as the average duration a subject remains in a specific CAP state before transitioning to another, was calculated to assess the stability of each state. The occurrence rate, representing the frequency of each CAP state relative to the total number of state occurrences, was computed to evaluate the prevalence of different states. Additionally, the transition probability matrix was derived, quantifying the likelihood of transitioning between different CAP states. To capture the complexity of state transitions, the entropy of Markov trajectories was calculated using the transition probability matrix (Ekroot and Cover 1993). This metric provides insight into the predictability and variability of state transitions, with lower entropy values indicating more deterministic transitions between CAP states (An et al. 2024).

### 2.7 Spatial Similarity and Transition Dynamics of CAP States

Spatial similarities of CAP states were quantified using Pearson correlation coefficients. Transition probabilities between CAPs were calculated from the observed state sequences. To evaluate the symmetry of transitions, forward and reverse transition probabilities for all state pairs were correlated. To investigate whether states with higher spatial similarity exhibit preferential transitions, the correlation between spatial similarity and symmetrized transition probabilities (average of the transition probability matrix and its transpose) was analyzed. This analysis was conducted at both group and individual levels, focusing on inter‐state transitions by excluding diagonal elements. The group‐level analysis employed the mean transition matrix across all subjects, while the individual‐level analysis considered subject‐specific transition matrices. For the individual‐level analysis, anti‐correlated CAP pairs were excluded to reduce their influence on linear fitting. These analyses aimed to elucidate the relationship between the spatial organization and temporal dynamics of brain states.

### 2.8 Reproducibility Analysis

To ensure the robustness of our findings, we conducted several complementary validation analyses (see Supporting Information for further details). First, we evaluated the influence of PCA dimensionality on clustering performance by comparing results using different numbers of principal components (8, 20, 60) and the full‐dimensional data. Second, we examined the stability of CAP dynamics across different cluster numbers (*k* = 6, *k* = 7, and *k* = 8) to verify that our main findings were not dependent on the specific choice of k. Third, we assessed whether deriving CAPs from different subject groups (healthy controls, schizophrenia patients, or all subjects combined) affected the detection of group differences. Fourth, we tested the stability of results across different parcellation schemes (AAL‐116, Brainnetome‐246, and Schaefer‐408). Finally, we performed external validation using the independent COBRE dataset to assess the generalizability of our findings.

### 2.9 Statistical Analysis

Demographic characteristics were compared using chi‐square tests for categorical variables and one‐way ANOVA for continuous variables. Clinical measures were compared between schizophrenia subgroups using independent t‐tests. Group comparisons of CAP dynamic features were conducted using analysis of covariance (ANCOVA), controlling for age, sex, and education level, with post hoc Tukey's HSD tests. Partial Spearman correlation analyses, adjusting for age, sex, and education level, examined relationships between CAP features and clinic symptom scores. Pearson correlation analyzed the relationship between spatial similarity and symmetrized transition probabilities of CAP states. All analyses were performed for schizophrenia subgroups (SCH_Neg, SCH_Non_Neg) and also the whole schizophrenia (SCH) group.

To control for multiple comparisons, false discovery rate (FDR) correction was performed using the Benjamini–Hochberg procedure. For comparisons between two groups (HC vs. SCH), FDR correction was applied across all features within each CAP dynamic property (e.g., spatial stability, temporal dynamics, transition probabilities). For analyses involving three groups (HC, SCH_Neg, SCH_Non_Neg), FDR correction was applied to the *p* values from post hoc pairwise tests (i.e., HC vs. SCH_Neg, HC vs. SCH_Non_Neg, SCH_Neg vs. SCH_Non_Neg) within each feature. For correlation analyses with clinical symptom scores, FDR correction was performed separately for each clinical scale within each CAP feature type. Statistical significance was set at *p* < 0.05 (two‐tailed). All statistical analyses were conducted using Python (version 3.10.11) with scipy (version 1.11.4), statsmodels (version 0.14.2), and pingouin (version 0.5.4) packages.

Additionally, to evaluate the potential influence of medication, we conducted sensitivity analyses by including chlorpromazine equivalent dosage as an additional covariate for participants with available medication information. A parallel analysis incorporating illness duration as a covariate was also performed. Further details of these sensitivity analyses are provided in the Supporting Information.

## 3 Results

### 3.1 Demographics and Clinical Characteristics

Table 1 summarizes the demographic and clinical characteristics of the study participants. Significant differences in education were observed among the three groups (HC, SCH_Neg, and SCH_Non_Neg groups) (*F* = 5.34, *p* = 0.006). SCH_Neg patients had higher PANSS negative scores (*t* = 4.78, *p* < 0.001), general psychopathology scores (*t* = 2.37, *p* = 0.021), and total PANSS scores (*t* = 2.92, *p* = 0.005) compared to SCH_Non_Neg patients.

**TABLE 1:** Demographic and clinical characteristics of the study participants.

Characteristic	HC (*n* = 34)	SCH_Neg (*n* = 31)	SCH_Non_Neg (*n* = 31)	Statistics	*p*
Sex (male, %)	21 (61.8%)	21 (67.7%)	22 (71.0%)	*χ*_(2)_^2^ = 0.64	0.725
Age	26.1 ± 3.0	24.6 ± 5.3	25.0 ± 5.9	*F*_(2.93)_ = 0.78	0.462
Year of education	14.9 ± 3.6	12.4 ± 2.9	13.2 ± 2.9	F_(2.93)_ = 5.34	0.006**
Duration of Illness	—	64.8 ± 42.8	56.3 ± 61.0 (*n* = 30)	*t*_(59)_ = 0.63	0.530
CPZ	—	211.5 ± 182.6 (*n* = 25)	239.0 ± 184.9 (*n* = 29)	*t*_(52)_ = −0.55	0.587
PANSS postive	—	12.5 ± 3.2	13.0 ± 5.3	*t*_(60)_ = −0.46	0.645
PANSS negative	—	27.4 ± 6.5	19.4 ± 6.6	*t*_(60)_ = 4.78	< 0.001***
PANSS general	—	38.6 ± 7.0	33.0 ± 11.1	*t*_(60)_ = 2.37	0.021*
PANSS total	—	78.5 ± 12.7	65.4 ± 21.5	*t*_(60)_ = 2.92	0.005**

***Note:*** Values are presented as mean ± standard deviation or *n* (%). Significance levels: *p < 0.05; **p < 0.01; ***p < 0.001.

Abbreviations: CPZ: chlorpromazine equivalent dose; HC: healthy controls; PANSS: Positive and Negative Syndrome Scale.; SCH_Neg: Schizophrenia patients with predominant negative symptoms; SCH_Non_Neg: Schizophrenia patients without predominant negative symptoms.

### 3.2 CAP States and Their Spatial Patterns

**FIGURE 1:**
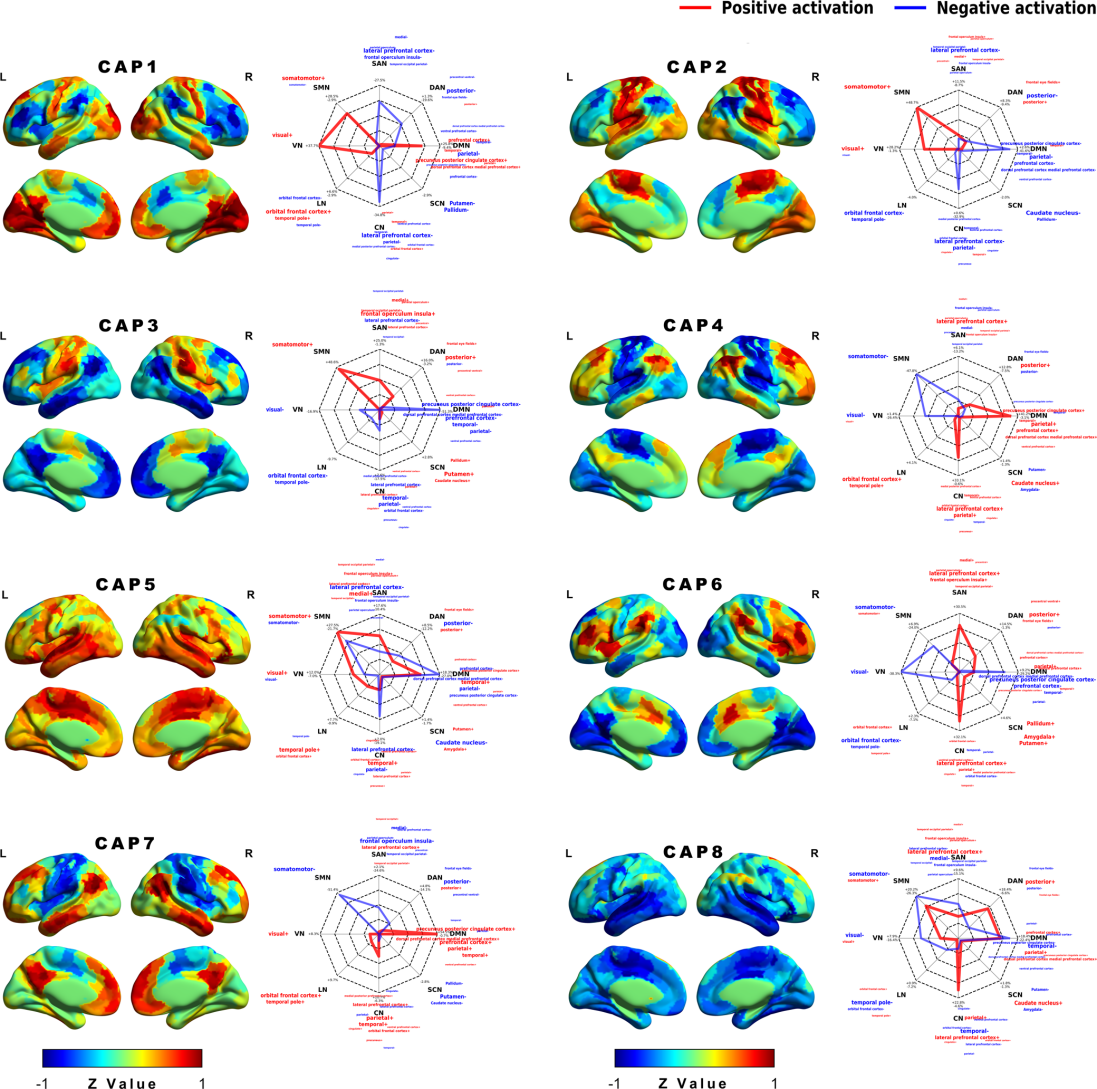
Spatial patterns of the eight identified CAPs. Each CAP represents a distinct configuration of positive (red) and negative (blue) activation across various brain networks. *Z* values indicate the degree of activation relative to the mean signal, with positive values representing increased activation and negative values representing decreased activation. Radar plots show the activation percentage for each network in each CAP, with connecting lines (red for positive activation, blue for negative activation) visualizing the overall activation pattern across brain networks. The CAPs are characterized as follows: CAP1 (VN+/SMN+/DMN+, CN‐/SAN‐), CAP2 (SMN+/VN+, DMN‐/CN‐), CAP3 (SMN+/SAN+, DMN‐/CN‐), CAP4 (DMN+/CN+, SMN‐/VN‐), CAP5 (SMN+, DMN‐/SMN‐), CAP6 (CN+/SAN+, VN‐/DMN‐/SMN‐), CAP7 (DMN+/CN+, SMN‐/SAN‐), and CAP8 (CN+/SMN+, SMN‐/DMN‐). Abbreviations: CN, control network; DAN, dorsal attention network; DMN, default mode network; LN, limbic network; SAN, salience/ventral attention network; SCN, subcortical network.; SMN, somatomotor network; VN, visual network.

We employed CAP analysis to explore dynamic co‐activation patterns across fMRI data, deriving coactivation patterns from all HC subjects through temporal k‐means clustering. Eight distinct CAP states were identified based on multiple evaluation metrics (see Figure S1), exhibiting unique spatial patterns of activation and deactivation across various functional networks. These states formed four pairs with opposing spatial configurations (Figure 1). To reflect the dominant network contributions, each CAP is described by its primary positively and negatively activated networks with activation percentages generally above 20%, ordered by activation strength. The first pair comprised CAP1 (VN+/SMN+/DMN+, CN‐/SAN‐) and CAP6 (CN+/SAN+, DMN‐/VN‐/SMN‐), while the second pair consisted of CAP2 (SMN+/VN+, DMN‐/CN‐) and CAP4 (DMN+/CN+, SMN‐/VN‐). The third pair included CAP3 (SMN+/SAN+, DMN‐) and CAP7 (DMN+/CN+, SMN‐/SAN‐), and the fourth pair contained CAP5 (SMN+, DMN‐/SMN‐) and CAP8 (SMN+/CN+, SMN‐/DMN‐), which share similar DMN deactivation patterns but show opposing network organization primarily through control network activity (prominent in CAP8 but minimal in CAP5) and through multiple other networks with reversed activation patterns (see Table S6 for complete network activation profiles). These anti‐correlated pairs underscored the dynamic interplay between different functional networks, particularly emphasizing the antagonistic relationships among the DMN, SMN, and SAN.

### 3.3 Transition Probabilities and Spatial Similarities Between CAP States

**FIGURE 2:**
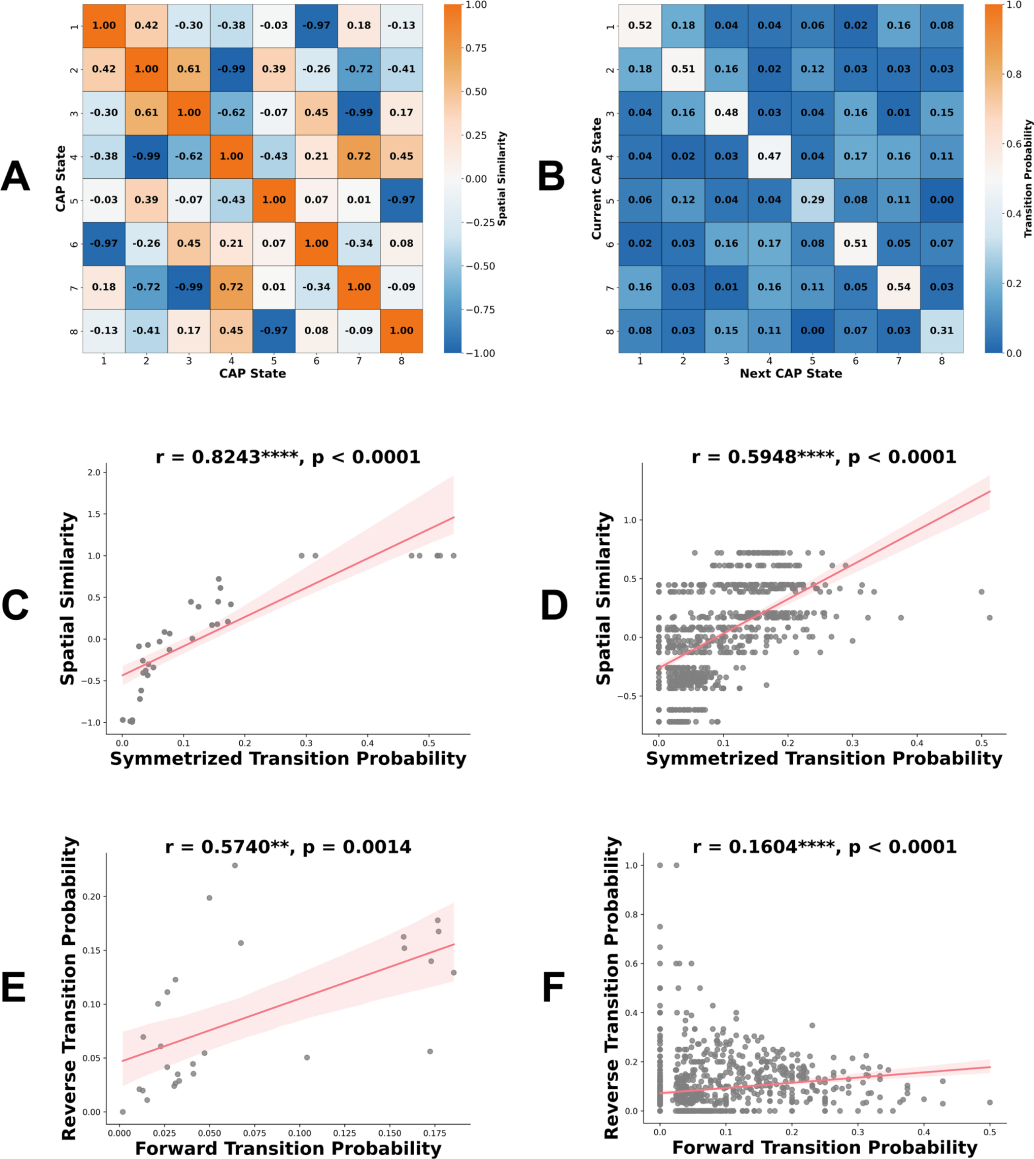
Spatial similarity and transition dynamics of CAP states in HC group. (A) Spatial similarity matrix between CAP states. (B) Group‐level transition probability matrix. (C) Group‐level correlation between spatial similarity and symmetrized transition probabilities. (D) Individual‐level correlation between spatial similarity and symmetrized transition probabilities, excluding anti‐correlated CAP pairs. (E) Group‐level transition probability symmetry analysis (y‐axis: CAP state B to A, x‐axis: CAP state A to B). (F) Individual‐level transition probability symmetry analysis (y‐axis: CAP state B to A, x‐axis: CAP state A to B). For C‐F, each point represents a CAP state pair, excluding diagonal elements (within‐state transitions). Shaded areas indicate 95% confidence intervals. Significance levels: *p < 0.05, **p < 0.01, ***p < 0.001, ****p < 0.0001.

Spatial similarity analysis identified four pairs of anti‐correlated CAP states (CAP1‐CAP6, CAP2‐CAP4, CAP3‐CAP7, CAP5‐CAP8), with correlation coefficients ranging from −0.97 to −0.99, demonstrating clear spatial symmetry within each pair (Figure 2A). The transition probability matrix revealed distinct patterns across states, with CAP7 and CAP1 showing the highest persistence probabilities (0.54 and 0.52, respectively), while CAP5 exhibited the lowest (0.29) (Figure 2B). Inter‐state transition probabilities were generally lower (0.01–0.18), with the most frequent transitions observed between CAP1‐CAP2 (0.18) and CAP4‐CAP6 (0.17). Notably, transitions between anti‐correlated CAP pairs were minimal (0.001–0.03), suggesting functional segregation among these pairs. Group‐level analysis showed a significant positive correlation between spatial similarity and symmetrized transition probabilities (*r =* 0.82, *p* < 0.001), which was also observed at the individual level (*r =* 0.59, *p* < 0.001) (Figure 2C,D). Examination of transition probability symmetry revealed positive correlations at both group (*r =* 0.57, *p* < 0.01) and individual (*r =* 0.16, *p* < 0.001) levels (Figure 2E,F), with a weaker correlation at the individual level, indicating asymmetry in dynamic transitions among CAP states. Additional analyses for SCH_Neg and SCH_Non_Neg groups are presented in the Figure S2.

### 3.4 Spatial Stability of CAP States

**FIGURE 3:**
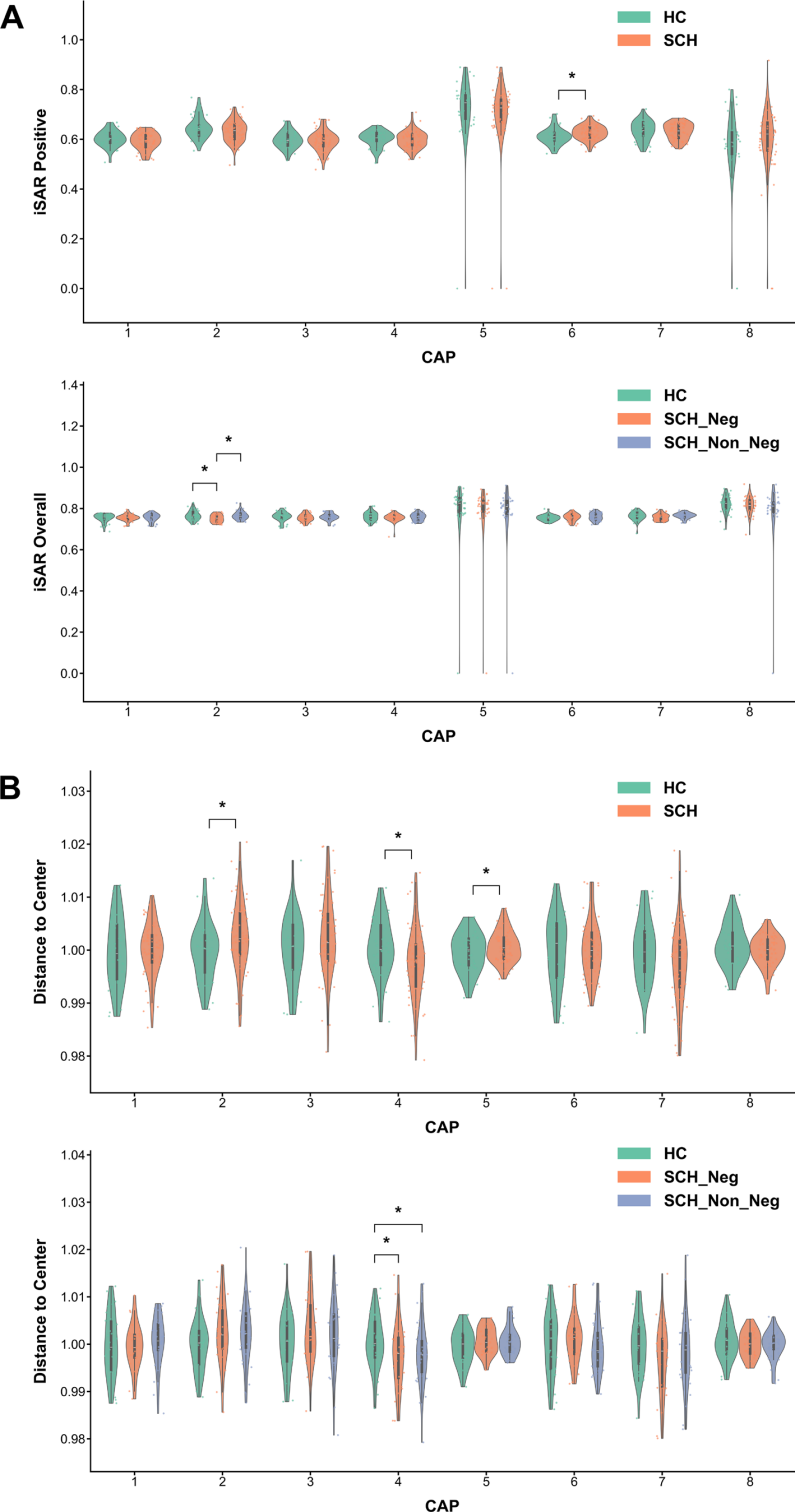
Spatial stability analysis of CAP states. (A) iSAR for each CAP state across groups. (B) Distance to CAP center for each state across groups. Asterisks indicate significant differences between groups (*p < 0.05, **p < 0.01, ***p < 0.001). Detailed statistical data are provided in Tables S1 and S2.

The spatial stability of CAPs revealed significant differences between groups. In the whole‐group comparison (HC vs. the whole SCH), schizophrenia patients exhibited greater distances to CAP centers in both CAP2 and CAP5, while showing reduced distance in CAP4 compared to HC, though these differences did not survive FDR correction (Figure 3A,B). Similarly, SCH patients showed higher positive iSAR in CAP6 compared to HC. In the subgroup comparison (HC, SCH_Neg, SCH_Non_Neg), CAP2 iSAR was lower in SCH_Neg compared to both HC and SCH_Non_Neg groups, while CAP4 distance to center was reduced in both patient subgroups compared to HC.

### 3.5 Temporal Dynamics of CAP States

**FIGURE 4:**
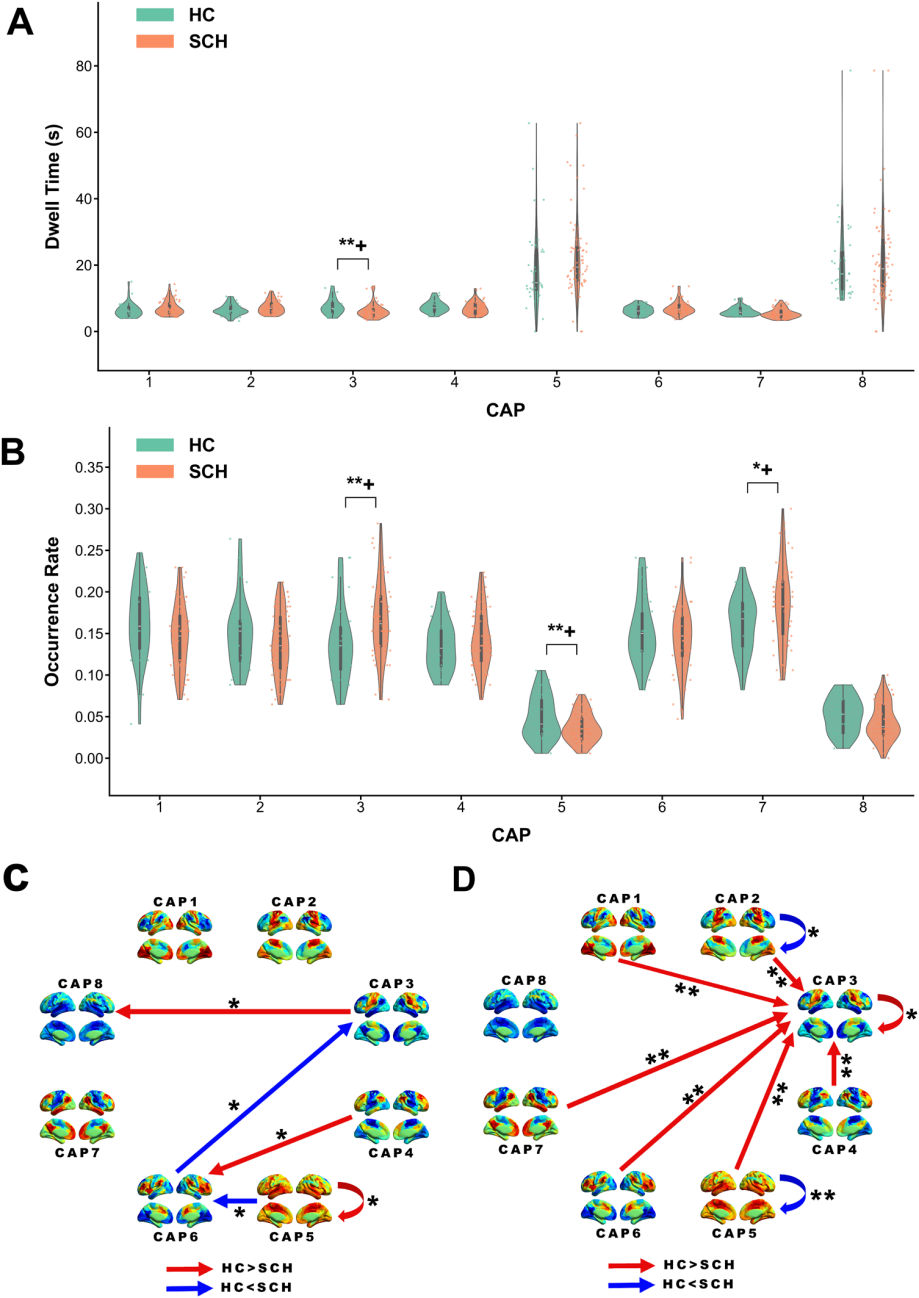
Temporal dynamics of CAP states in two‐group comparison (HC vs. SCH). (A) Dwell time for each CAP state. (B) Occurrence rate for each CAP state. (C) Transition probabilities between CAP states. (D) Markov trajectory entropy for transitions between CAP states. Bar plots show mean values with standard error. Asterisks indicate significant differences between groups (*p < 0.05, **p < 0.01, ***p < 0.001), while + indicates significance retained after FDR correction applied separately within each category of CAP dynamic properties. Detailed statistical data are provided in Table S1.

The temporal dynamics of CAP state differed significantly among groups. In the whole‐group comparison (HC vs. the whole SCH; Figure 4, Table S1), CAP3 exhibited longer dwell times in HC compared to SCH (*p* = 0.006) (Figure 4A). Occurrence rates differed significantly for CAP3 (*p* = 0.007), CAP5 (*p* = 0.006), and CAP7 (*p* = 0.019) (Figure 4B). Specifically, SCH showed higher occurrence rates for CAP3 and CAP7 compared to HC, while HC exhibited a higher rate for CAP5. Transition probability analysis revealed differences in several transitions, with HC showing higher probabilities for CAP3 to CAP8, CAP4 to CAP6, and CAP5 self‐transitions, while SCH exhibited higher probabilities for CAP5 to CAP6 and CAP6 to CAP3 transitions (Figure 4C). Markov trajectory entropy was higher in HC for transitions to CAP3 from multiple states and lower for CAP2 and CAP5 self‐transitions compared to SCH (Figure 4D).

**FIGURE 5:**
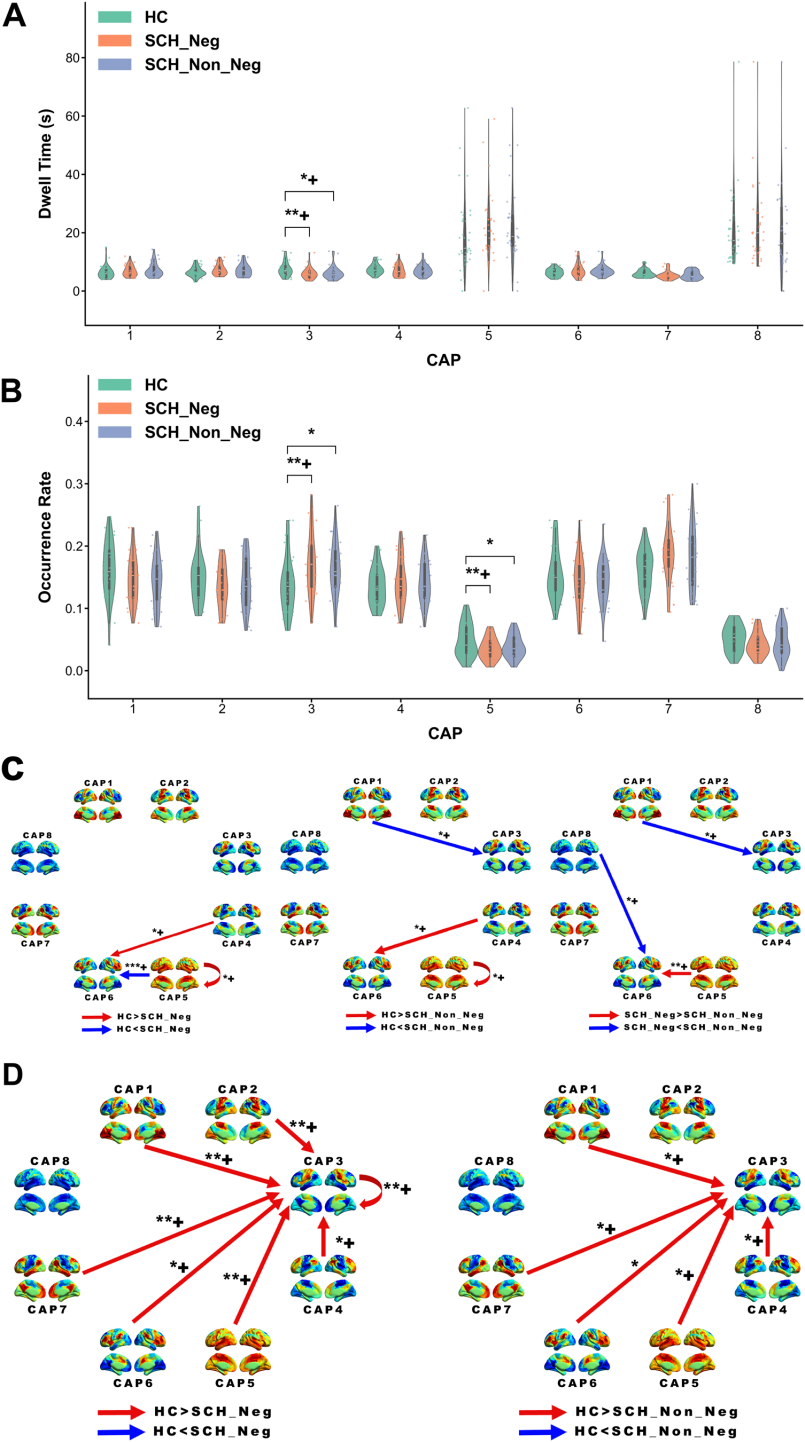
Temporal dynamics of CAP states in three‐group comparison (HC, SCH_Neg, SCH_Non_Neg). (A) Dwell time for each CAP state. (B) Occurrence rate for each CAP state. (C) Transition probabilities between CAP states. (D) Markov trajectory entropy for transitions between CAP states. Bar plots show mean values with standard error. Asterisks indicate significant differences between groups (*p < 0.05, **p < 0.01, ***p < 0.001), while + indicates significance retained after FDR correction applied to post hoc pairwise tests within each CAP state. Detailed statistical data are provided in Table S2.

In the subgroup comparison (HC, SCH_Neg, SCH_Non_Neg; Figure 5, Table S2), both patient groups exhibited shorter CAP3 dwell times compared to HC, with SCH_Neg showing the most pronounced reduction (Figure 5A). For occurrence rates, SCH_Neg patients showed higher CAP3 rates and lower CAP5 rates relative to HC (Figure 5B). Regarding transition probabilities, SCH_Non_Neg exhibited increased CAP1 to CAP3 transitions compared to both HC and SCH_Neg groups, while HC demonstrated higher probabilities in CAP4 to CAP6 and CAP5 self‐transitions compared to both patient groups. Most notably, SCH_Neg patients showed distinct CAP5 to CAP6 transition patterns, with significantly higher probabilities than both HC and SCH_Non_Neg groups (Figure 5C). Markov trajectory entropy analysis revealed lower entropy values in both patient groups compared to HC across multiple transitions to CAP3, with SCH_Neg showing the most consistent reductions (Figure 5D).

### 3.6 Clinical Correlations of CAP Features

Significant correlations between CAP features and clinical symptoms were observed in the whole SCH group after FDR correction. The distance to center for CAP7 positively correlated with PANSS positive scores (*r =* 0.343, *p =* 0.032), while the distance to center for CAP3 showed a significant negative correlation with positive symptoms (*r =* −0.366, *p =* 0.032). The occurrence rate of CAP8 was positively associated with PANSS positive (*r =* 0.430, *p =* 0.005), general psychopathology (*r =* 0.449, *p =* 0.003), and total scores (*r =* 0.443, *p =* 0.004). For transition probabilities, CAP5 to CAP7 transitions positively correlated with PANSS positive scores (*r =* 0.438, *p =* 0.032), while CAP3 to CAP1 transitions showed strong negative correlations with negative symptoms (*r =* −0.528, *p =* 0.001), general psychopathology (*r =* −0.493, *p =* 0.005), and total scores (*r =* −0.526, *p =* 0.001). These correlations are visualized in Figure S5.

**FIGURE 6:**
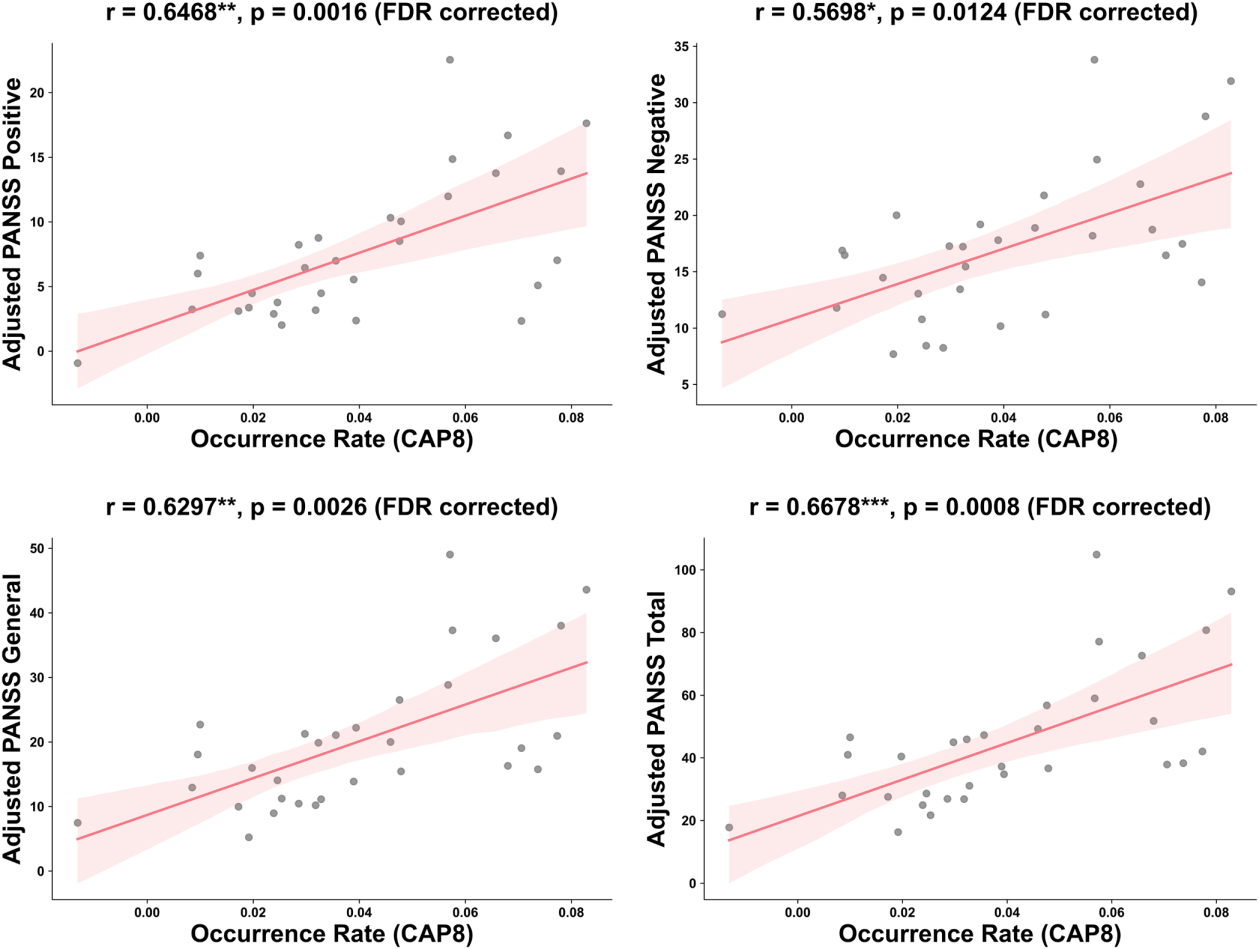
Significant correlations between CAP features and clinical symptoms in the SCH_Non_Neg group. Only correlations that remained significant after FDR correction are shown. FDR correction was applied separately for each clinical scale within each CAP feature. Significant levels: *p < 0.05, **p < 0.01, ***p < 0.001 (FDR‐corrected).

Further analysis of SCH subgroups revealed distinct patterns. In the SCH_Neg group, no significant correlations remained after FDR correction (Figure S6). In contrast, the SCH_Non_Neg group showed strong positive correlations between the occurrence rate of CAP8 and all PANSS measures after FDR correction: positive symptoms (*r =* 0.647, *p =* 0.002), negative symptoms (*r =* 0.570, *p =* 0.012), general psychopathology (*r =* 0.630, *p =* 0.003), and total score (*r* = 0.668, *p =* 0.001) (Figures 6 and S7). The different correlation patterns between subgroups suggest distinct neurobiological mechanisms may underlie symptom manifestation in different schizophrenia subtypes.

### 3.7 Reproducibility of CAP Analysis

To assess the robustness of our findings, we conducted comprehensive reproducibility analyses across multiple methodological dimensions (detailed in Supporting Information). Varying the PCA dimensionality (8, 20, 60 components, and full data) revealed consistent spatial patterns (*r =* 0.98–1.0) and maintained key group differences in temporal dynamics across all conditions (Figure S8). Comparing different cluster numbers (*k* = 6, 7, 8) demonstrated high spatial correspondence between equivalent CAP states (*r =* 0.68–0.95) and preserved significant group differences, particularly for occurrence rates of corresponding states (Figure S9). Analysis of different clustering sources (HC, SCH, or combined) showed that HC‐derived CAPs provided optimal sensitivity for detecting disease‐related alterations while maintaining consistent spatial patterns (Figure S10). Testing across multiple brain parcellation schemes (AAL‐116, Brainnetome‐246, Schaefer‐408) confirmed that our findings were robust to atlas choice, with the Schaefer‐408 atlas providing superior clustering quality (Figure S11). Finally, external validation using the independent COBRE dataset demonstrated strong spatial correspondence between matched CAP states (*r* > 0.90) and replicated key group differences in temporal dynamics in a two‐group comparison (HC vs. SCH) (Figure S12).

### 3.8 Medication and Illness Duration Effects

Sensitivity analyses examining the influence of antipsychotic medication and illness duration revealed differential effects on CAP features. In the medication model, none of the previously significant features in the two‐group comparison retained significance, while in the subgroup comparison, only the difference in CAP2 iSAR between SCH_Neg and SCH_Non_Neg remained significant (*t* = 2.457, *p =* 0.016). The illness duration model preserved more significant findings, including CAP5 occurrence rate (*F* = 6.077, *p =* 0.016) and multiple Markov trajectory entropy measures in the two‐group comparison. In the subgroup analysis, the duration model maintained significant differences in CAP2 iSAR (HC vs. SCH_Neg, *t* = −2.25, *p =* 0.027; SCH_Neg vs. SCH_Non_Neg, *t* = 2.323, *p =* 0.022), CAP5 occurrence rate (HC vs. SCH_Neg, *t* = −2.56, *p* = 0.012), and CAP5 to CAP6 transition probability (HC vs. SCH_Neg, *t* = 2.187, *p* = 0.031; SCH_Neg vs. SCH_Non_Neg, *t* = −2.756, *p* = 0.007). Detailed results are presented in Tables S4 and S5.

## 4 Discussion

In this study, we investigated dynamic network characteristics in schizophrenia using CAP analysis, focusing on patients with predominant negative symptoms. Our findings revealed two distinct layers of network disruption: general disease effects and symptom‐specific alterations. At the general disease level, patients exhibited altered spatial stability, with tendencies towards greater variability in sensorimotor‐related networks (CAP2, CAP5) and reduced variability in a default‐mode state (CAP4). Temporally, they displayed instability in a key sensorimotor‐salience state (CAP3), evidenced by decreased dwell time and increased occurrence, alongside a reduced prevalence of a sensorimotor‐dominant state (CAP5). At the symptom‐specific level, the SCH_Neg group was uniquely characterized by significantly lower spatial stability in a sensorimotor‐visual state (CAP2). This group also showed profound temporal disruptions, including a sharply reduced CAP3 dwell time, lower CAP5 occurrence, and markedly increased transitions from this sensorimotor state to a control‐salience state (CAP5 to CAP6). The dissociation between global disease‐related changes and subgroup‐specific patterns underscores the heterogeneity of neural dysfunction in schizophrenia.

### 4.1 Neural Mechanisms Underlying Spatial Stability Alterations

The observed network stability alterations in schizophrenia reveal distinct patterns of disruption in both regional activation consistency and global spatial configurations, manifesting general disease effects and symptom‐specific characteristics (Doucet et al. 2020; Georgiadis et al. 2024). At the general disease level, schizophrenia patients demonstrated impaired stability in maintaining sensorimotor network states, evidenced by increased spatial variability (greater distance to center) in both CAP2 (sensorimotor‐visual) and CAP5 (sensorimotor‐dominant). Particularly noteworthy was the disrupted balance in the anti‐correlated CAP2‐CAP4 pair, where schizophrenia patients showed destabilized sensorimotor‐visual processing in CAP2 coupled with an overly rigid default‐control state (CAP4), in contrast to the balanced stability maintained by healthy controls. This imbalanced pattern, together with the destabilization of the sensorimotor‐dominated CAP5, suggests a fundamental disruption in the brain's ability to maintain appropriate dynamic segregation between external sensory and internal cognitive processing (Fan et al. 2022; Kaufmann et al. 2015; Long et al. 2020).

Crucially, the destabilization of the sensorimotor‐visual state (CAP2) was most pronounced in the SCH_Neg group, which showed significantly lower regional activation consistency (iSAR) compared to both controls and SCH_Non_Neg patients. This suggests that while a general deficit in sensorimotor network stability exists in schizophrenia, a profound and specific inability to consistently engage sensorimotor‐visual networks for environmental processing may be a distinct neural signature of predominant negative symptoms (Kaufmann et al. 2015; Sun et al. 2021). In contrast, the enhanced stability of the control‐salience network (increased positive iSAR in CAP6) in the overall patient group may hint at a compensatory mechanism or a different aspect of network dysregulation that warrants further investigation (Arkin et al. 2020; Horne et al. 2021). Taken together, while global spatial stability alterations in several states characterize the disease process, the specific and robust destabilization of CAP2 in the SCH_Neg group points toward a key mechanism underlying symptom heterogeneity in schizophrenia (Pico‐Perez et al. 2022; Vanes et al. 2019; Voineskos et al. 2024), where preserved sensorimotor integration might serve as a protective factor against negative symptoms.

### 4.2 Temporal Dynamics and Clinical Implications

Analysis of temporal dynamics revealed distinct alterations in brain network states across schizophrenia patients. At the general disease level, the shortened dwell time of CAP3 (sensorimotor‐salience) indicates reduced temporal stability in coordinating sensorimotor and salience network activation while suppressing default mode activity (Kottaram et al. 2019), consistent with widespread temporal stability reduction in sensory and perceptual systems observed in schizophrenia (Hegarty et al. 2020; Hou et al. 2023; Long et al. 2023). The altered occurrence pattern further suggests a shift from external sensory processing to internal cognitive states, marked by an increased occurrence of the DMN‐control state (CAP7) at the expense of the sensorimotor‐dominant state (CAP5) (Bolton et al. 2020; Dima et al. 2020; Yang, Tang, et al. 2021; Yang, Zhang, et al. 2021). These temporal characteristics manifest through complex alterations in state transition patterns. Notably, patients exhibited heightened transitions from the sensorimotor‐dominant state (CAP5) to the control‐salience state (CAP6), while showing reduced transitions between other states like CAP4 to CAP6. Further, the flexibility of network dynamics showed a multifaceted dysregulation; pathways leading into the sensorimotor‐salience state (CAP3) became more rigid and predictable (lower entropy), whereas the ability to sustain certain perceptual states appeared compromised (higher entropy for CAP2 and CAP5 self‐transitions), reflecting a challenged ability to maintain flexible network configurations necessary for adaptive behavior (Blair et al. 2024; Hou et al. 2023; Wang, Peng, et al. 2021; Wang, Jiang, et al. 2021; Yang et al. 2015). The alterations in CAP3 temporal dynamics, together with the overall pattern of network state changes, point to disrupted integration between large‐scale brain networks (Snyder et al. 2021; Wang et al. 2016; Yang, Tang, et al. 2021; Yang, Zhang, et al. 2021), underlying impaired sensory processing and environmental interaction (Long et al. 2023; Schimmelpfennig et al. 2023).

SCH_Neg patients showed distinct temporal characteristics that suggest specific pathophysiological mechanisms (Giuliani et al. 2024; Raucher‐Chene et al. 2022; Wang, Chang, and Wang 2023). This was most evident in their state transition dynamics, where the SCH_Neg group exhibited a markedly increased probability of transitioning from the sensorimotor state (CAP5) to the control‐salience state (CAP6). This suggests a profound inability to sustain engagement with sensorimotor information, leading to an excessively rapid, and perhaps premature, recruitment of higher‐order control networks. Such an imbalanced dynamic—an impaired maintenance of perceptual states coupled with a hyperactive switch to cognitive control—may directly underlie the avolition and diminished expression characteristic of negative symptoms (Cattarinussi et al. 2023; Correll and Schooler 2020; Wertz et al. 2019). This interpretation is supported by converging evidence from our data: this group also showed the most pronounced reduction in CAP3 dwell time and the lowest occurrence of CAP5, alongside the most consistently reduced entropy for transitions into CAP3, all pointing to a core deficit in maintaining stable, externally focused brain states. In contrast, the SCH_Non_Neg group displayed a different pattern, with increased transitions from CAP1 to CAP3, potentially reflecting a more adaptive dynamic that preserves a degree of flexibility between perceptual and cognitive states (Collin et al. 2025; Kuehn et al. 2025). It is important to acknowledge, however, that the SCH_Neg group also presented with higher general psychopathology scores. While the observed temporal patterns strongly align with the negative symptom profile, future studies should aim to disentangle the unique contribution of negative symptoms from that of overall illness severity (Chan et al. 2022; Voineskos et al. 2024). These distinct temporal characteristics not only advance our understanding of symptom‐specific mechanisms but also provide potential neuroimaging markers for clinical assessment and therapeutic targeting (Giuliani et al. 2024; Ramirez‐Mahaluf et al. 2023; Wang, Zhang, et al. 2023).

### 4.3 Clinical Correlations and Symptom‐Specific Mechanisms

Our correlation analyses revealed complex relationships between network dynamics and clinical symptomatology, highlighting both pathological and potentially adaptive neural processes. At the general disease level, network instability manifested through two primary patterns. First, states associated with internal mentation and higher‐order control were linked to greater symptom severity. The instability of CAP7 (DMN‐control) and higher occurrence of CAP8 (cognitive‐sensorimotor integration) correlated with more severe positive and general psychopathology, respectively (Yang, Tang, et al. 2021; Yang, Zhang, et al. 2021; You et al. 2022). This suggests that excessive engagement of these internal cognitive states may contribute to clinical symptoms (Kottaram et al. 2019). Second, and perhaps more importantly, we identified a potentially protective dynamic: a higher probability of transitioning from CAP3 (sensorimotor‐salience) to CAP1 (integrated visual‐sensorimotor‐DMN) was strongly associated with lower negative and general symptom scores. This suggests that the ability to efficiently transition from processing salient external information to a state of broader perceptual and cognitive integration may serve as a crucial resilience factor against core symptoms of schizophrenia (Bolton et al. 2020; Hare et al. 2019; Thakuri et al. 2024).

The subgroup analysis revealed two distinct clinical‐neural profiles, clearly illustrating the heterogeneity of the disorder. Notably, the SCH_Neg group exhibited no significant correlations between their pronounced network alterations and symptom severity. This dissociation provides strong support for a “trait‐like” model of predominant negative symptoms, where the observed network dysfunctions (e.g., CAP2 instability and rapid CAP5‐to‐CAP6 transitions) represent a stable, enduring neurobiological characteristic rather than a fluctuating state‐dependent phenomenon (Giordano et al. 2023; Raucher‐Chene et al. 2022; Wu et al. 2021). In stark contrast, the SCH_Non_Neg group displayed a dynamic, state‐dependent symptom‐network relationship. Here, the occurrence rate of CAP8 correlated strongly with all dimensions of symptomatology, including positive, negative, and general psychopathology. This finding is critical as it directly addresses the potential confounding influence of general symptoms, suggesting that in this subgroup, CAP8 occurrence may function as a state‐dependent marker of overall illness severity rather than a mechanism specific to one symptom domain (Li et al. 2023; Niu et al. 2025). These distinct patterns demonstrate that predominant negative symptoms may arise from a stable disruption of network flexibility, whereas other symptoms in less severe subtypes could emerge from state‐dependent network dysregulations that track with overall illness burden (Bolton et al. 2020; Vanes et al. 2019; Yang, Tang, et al. 2021; Yang, Zhang, et al. 2021).

### 4.4 Medication and Illness Duration Effects

A critical consideration in schizophrenia neuroimaging is the potential confounding influence of antipsychotic medication and illness duration. Our sensitivity analyses revealed differential effects of these factors on CAP features. When controlling for chlorpromazine equivalent dosage, most group differences observed in our primary analyses, particularly in the two‐group comparison, did not retain statistical significance. This outcome may suggest that some observed network dynamics are influenced by medication, or alternatively, that antipsychotic treatment may partially normalize or mask underlying pathophysiological alterations, a distinction our cross‐sectional design cannot fully resolve (Sarpal et al. 2015; Wang, Peng, et al. 2021; Wang, Jiang, et al. 2021; Yang, Tang, et al. 2021; Yang, Zhang, et al. 2021). Notably, the reduced regional stability (iSAR) of CAP2 robustly differentiated the SCH_Neg group from the SCH_Non_Neg group even after accounting for medication effects. This provides strong support that this specific deficit in sensorimotor‐visual network stability is a core feature of the predominant negative symptom subtype, potentially independent of typical medication exposure.

In contrast, a greater number of findings remained significant after controlling for illness duration. Crucially, the key temporal signatures characterizing the SCH_Neg group—including their lower CAP5 occurrence rate and, most importantly, their heightened transition probability from CAP5 to CAP6—remained robust. The resilience of these findings to the effects of illness duration argues against them being merely a consequence of disease chronicity. Instead, it strengthens the interpretation that these specific temporal dynamics, particularly the excessive switching from sensorimotor to control‐salience states, may represent stable, trait‐like neural markers of the negative symptom phenotype rather than an artifact of long‐term illness (Li et al. 2025; Li et al. 2017). Taken together, while caution is warranted in interpreting findings sensitive to medication, our sensitivity analyses underscore the robustness of the core spatial (CAP2 iSAR) and temporal (CAP5‐to‐CAP6 transition) features that distinguish patients with predominant negative symptoms, bolstering their potential as valid neurobiological targets.

### 4.5 Strengths and Limitations

The primary strength of this study lies in its comprehensive approach to ensuring the robustness and generalizability of our findings. Our CAP analysis results are consistent with established principles of brain network dynamics, such as the preferential transitions between spatially similar states and the functional segregation of anti‐correlated networks, lending confidence to our methodological approach (Sun et al. 2024; Yang, Tang, et al. 2021; Yang, Zhang, et al. 2021; Zhang et al. 2024). Crucially, we conducted extensive validation analyses, detailed in the Supporting Information, which demonstrated the stability of our main findings across different analytical choices, including PCA dimensionality, cluster numbers, and parcellation schemes. Furthermore, the successful replication of key temporal dynamic alterations in the independent COBRE dataset substantially strengthens the generalizability of our results. The three‐group design was another key strength, enabling the dissociation of general disease‐related effects from neural patterns specific to predominant negative symptoms.

Nevertheless, several limitations should be acknowledged. First, the sample size is relatively modest, and the single‐center design may limit the broader generalizability of our findings. Second, while our sensitivity analyses addressed the influence of medication and illness duration, the cross‐sectional nature of the data precludes definitive conclusions about their causal effects. The observed group differences in general psychopathology scores also warrant caution in attributing all findings exclusively to negative symptomatology. Finally, the temporal resolution of our fMRI data (TR = 2.6 s) may not fully capture more rapid state transitions. Future research employing larger, multi‐center, and longitudinal cohorts is needed to confirm these findings, track their developmental trajectory, and ultimately explore their potential as targets for therapeutic intervention.

### 4.6 Conclusions

Our analysis revealed distinct patterns of dynamic network disruption in schizophrenia, particularly in relation to negative symptoms. SCH_Neg patients exhibit impaired sensorimotor‐visual network stability and excessive transitions from sensorimotor to control‐salience states. In addition, these network alterations are independent of symptom severity, suggesting stable trait‐like reorganization. In contrast, SCH_Non_Neg patients maintain more adaptive network dynamics, with state‐dependent dysregulation that correlates with clinical symptoms, potentially serving as a buffer against negative symptomatology. These findings advance our understanding of schizophrenia's neural underpinnings and provide a foundation for developing targeted interventions based on dynamic network biomarkers.

## Conflicts of Interest

The authors declare no conflicts of interest.

## Supporting information


**Data S1:** Supporting Information.

## Data Availability

The analysis code used in this study is publicly available at https://github.com/hisonWarren/co‐activation‐pattern‐analysis.git. The neuroimaging data that support the findings of this study are available from the corresponding author upon reasonable request. However, the data are not publicly available due to privacy concerns and ethical restrictions imposed by the institutional ethics committee.
