# Supplementary material for: Altered Brain Network Dynamics in Schizophrenia Patients With Predominant Negative Symptoms: A Resting‐State fMRI Study Using Co‐Activation Pattern Analysis
Source: Hum Brain Mapp. 2025 Sep 24;46(14):e70369. doi: 10.1002/hbm.70369 (PMC12460708; doi:10.1002/hbm.70369)
Supplement: Supplementary file 1 — Data S1: Supporting Information. [file HBM-46-e70369-s001.docx]

Supplementary Materials

### 1. Clustering Evaluation

The optimal number of co-activation pattern (CAP) states was determined through comprehensive evaluation of multiple clustering metrics. Fig.S1 presents the results of six key evaluation metrics across different numbers of clusters: Sum of Squared Errors (SSE), Silhouette Score (Rousseeuw, 1987), Calinski-Harabasz Index (Caliński & and Harabasz, 1974), Davies-Bouldin Index (Davies & Bouldin, 1979), Average Transition Probability, and Mean Error. These metrics were systematically calculated for cluster solutions ranging from 2 to 20, providing a comprehensive assessment of both cluster quality and temporal stability.

The SSE and Mean Error metrics quantify within-cluster compactness by measuring the squared and linear distances, respectively, between each time point and its assigned cluster center. Lower values of both metrics indicate tighter, more homogeneous clusters. The Silhouette Score evaluates both cluster cohesion and separation by comparing each point's similarity to its own cluster versus other clusters, with values ranging from -1 to 1, where higher scores indicate better-defined clusters. The Calinski-Harabasz Index assesses cluster validity through the ratio of between-cluster to within-cluster variance, with higher values indicating greater separation between clusters relative to their internal variation. The Davies-Bouldin Index measures the average similarity between each cluster and its most similar cluster, with lower values representing better separation between distinct clusters. The Average Transition Probability metric was calculated from the state transition matrix, representing the likelihood of transitioning between different brain states. Lower transition probabilities indicate greater temporal stability of the identified states, a critical neurophysiological consideration unique to time series-based brain state analyses.

While individual metrics showed varying trends, a holistic examination of all metrics suggested that solutions with 6 to 8 clusters provided a good balance between model complexity and data fit (Fig. S1). This range aligns with previous studies in the field, which have typically identified 6 to 8 distinct CAP states (An et al., 2024; Janes et al., 2020; Sun et al., 2024; Yang et al., 2021). Considering the specific characteristics of the current dataset and the need for a comprehensive representation of brain co-activation patterns, 8 clusters were selected as the optimal solution for this study.

**2. Calculation of Spatial Stability Metrics**

**(1) Individual Stable Activation Rate (iSAR)**

Individual Stable Activation Rate (iSAR) quantifies the consistency of activation patterns within each CAP state at the subject level. For CAP state ***k*** and brain region ***r***, the **stable regions** are defined as those with normalized activation exceeding a threshold**θ= 0.4**:

**Stable positive regions**:

$$S_{k}^{+}=\left\{ r | Z_{k,r}>\theta\right\}$$

**Stable negative regions**:

$$S_{k}^{-}=\left\{ r | Z_{k,r}<-\theta\right\}$$

Where $Z_{k,r}$is the normalized activation of region ***r*** in CAP state ***k***.

The threshold **θ=0.4** was selected based on prior studies (Zhang et al., 2024), aiming to balance sensitivity and specificity.

The iSAR quantifies the proportion of frames that maintain stable activation in the identified regions:

**Stable positive iSAR**:

$$\text{iSAR}_{k}^{+}=\frac{1}{\left| F_{k} \right|}\sum_{f\in F_{k}} \frac{\left| \left\{ r\in S_{k}^{+} | Z_{f,r}>\theta\right\} \right|}{\left| S_{k}^{+} \right|}$$

**Stable negative iSAR**:

$$\text{iSAR}_{k}^{-}=\frac{1}{\left| F_{k} \right|}\sum_{f\in F_{k}} \frac{\left| \left\{ r\in S_{k}^{-} | Z_{f,r}<-\theta\right\} \right|}{\left| S_{k}^{-} \right|}$$

**Overall iSAR**:

$$\text{iSAR}_{k} = \frac{1}{\left| F_{k} \right|} \sum_{f \in F_{k}} \frac{\left| \left\{ r \in S_{k}^{+} \cup S_{k}^{-} \mid\left| Z_{f,r} \right| > \theta\right\} \right|}{\left| S_{k}^{+} \cup S_{k}^{-} \right|}$$

where:

$F_{k}$ is the set of frames assigned to CAP state $k$,

$\mid\cdot\mid$enotes set cardinality.

Higher iSAR values indicate greater spatial stability in maintaining the characteristic spatial configuration of the CAP state.

**(2) Distance to Center**

This metric quantifies spatial variability of each frame with respect to the canonical center of its assigned CAP state. For a frame ***f*** assigned to state ***k***, the **distance to center** is defined as:

$$D_{f,k}=1-r\left( f,C_{k} \right)$$

where$r\left( f,C_{k} \right)$ is the Pearson correlation coefficient between frame *f* and the center pattern ***C_k​_*** of state ***k***.

The **mean distance** across all frames in CAP state ***k*** is:

$$\bar{D}_{k}=\frac{1}{\left| F_{k} \right|}\sum_{f\in F_{k}} D_{f,k}$$

Lower $\bar{D}_{k}$ values indicate greater spatial stability, reflecting consistent activation patterns that closely resemble the CAP state prototype.

**3. Calculation of Temporal Properties**

**(1) Dwell Time**

Dwell time quantifies the average temporal duration a given CAP state persists before switching. For CAP state *k*, dwell time $D_{k}$is defined as:

$$D_{k}=\frac{T_{k}}{N_{k}}$$

where $T_{k}$ is the total number of time points assigned to state k, and $N_{k}$ is the number of occurrences of state *k* across the time series. This metric reflects the temporal stability of individual brain states.

**(2) Occurrence Rate**

Occurrence rate measures the relative frequency of each CAP state's appearance. For state k, the occurrence rate $O_{k}$ is computed as:

$$O_{k}=\frac{N_{k}}{N_{\text{total}}}$$

where $N_{k}$ is the number of transitions into state k, and $N_{total}$ is the total number of CAP occurrences in the time series. This metric represents the overall prevalence of each state.

**(3) Transition Probability Matrix**

The transition probability matrix $P\in\mathbb{R}^{K\times K}$describes the empirical probability of switching between CAP states. Each element $P_{ij}$is defined as:

$$P_{ij}=\frac{n_{ij}}{\sum_{j} n_{ij}}$$

where $n_{ij}$ is the number of observed transitions from state i to state j. Diagonal elements $P_{ii}$ represent persistence probabilities; off-diagonal values indicate transition dynamics between distinct states.

**(4) Symmetrized Transition Matrix**

To eliminate directionality in transition analysis, the symmetrized transition matrix is computed as:

$$P_{\text{sym}}=\frac{1}{2}\left( P+P^{\top} \right)$$

where *P* is the transition probability matrix and *P*^T^ its transpose. This representation captures bidirectional transition strength between CAP states while preserving overall transition structure.

**(5) Entropy of Markov Trajectories**

The entropy of Markov trajectories measures the uncertainty associated with state transitions in a stationary Markov chain defined by transition matrix$\boldsymbol{P}$ and stationary distribution $\boldsymbol{\mu}$ (Ekroot & Cover, 1993). The entropy rate $H(X)$ is given by:

$$H\left( X \right)=-\sum_{i,j} \mu_{i}P_{ij}\log P_{ij}$$

where the stationary distribution satisfies:

$$\mu_{j}=\sum_{i} \mu_{i}P_{ij}$$

The entropy matrix $\boldsymbol{H}$of trajectories from state i to j is computed as:

$$\boldsymbol{H}=\boldsymbol{K-}\tilde{\boldsymbol{K}}+\text{diag}\left( H_{ii} \right)$$

with components defined by:

- $H_{ii}=\frac{H\left( X \right)}{\mu_{i}}$
- ${H_{i}}^{*}=-\sum_{j} P_{ij}\log P_{ij}$
- $\boldsymbol{K}=\left( \boldsymbol{I-P+A} \right)^{-1}\left( diag(H^{*})-{diag(H}_{ii} \right))$
- $\tilde{K}_{ij}=K_{jj}$
- $A_{ij}=\mu_{j}$

where $\boldsymbol{I}$is the identity matrix, and diag(⋅) denotes a diagonal matrix formed from the vector argument.

Lower entropy values indicate more deterministic transitions with higher predictability, while higher entropy values reflect increased variability and uncertainty in CAP state transitions.

**4 Reproducibility analysis**

**4.1 PCA Dimensionality Reduction Analysis**

We investigated the impact of PCA dimensionality reduction on our clustering results by comparing multiple conditions: 8, 20, and 60 components, as well as the full 408-dimensional data. Across all conditions, clustering evaluation metrics consistently identified the optimal number of CAP states between 6 and 8. The 8-component PCA solution demonstrated superior performance with the highest Silhouette Score and best Davies-Bouldin Index, while maintaining comparable Calinski-Harabasz Index values (Fig. S8A). Importantly, spatial correlation analysis revealed high consistency between corresponding CAP states across dimensionality reduction approaches (r=0.98-1.0 for matched pairs) (Fig. S8B). Critical to our findings, the significant group differences in temporal dynamics remained robust across all PCA conditions (Fig. S8C).

**4.2 Cluster Number Reproducibility Analysis**

To assess whether our findings remained consistent across different clustering solutions, we systematically compared CAP states derived using k=6, k=7, and k=8. Spatial patterns of CAP states demonstrated consistent network configurations across different k values (Fig. S9A). Correlation analysis between k=8 CAPs and those derived from k=6 and k=7 revealed high spatial correspondence (correlation coefficients ranging from 0.68 to 0.95) (Fig. S9B). The occurrence rate of CAP5 (k=8) showed significant group differences in both two-group (HC vs. SCH, p < 0.01) and three-group comparisons (HC vs. SCH_Neg [schizophrenia patients with predominant negative symptoms, SCH_Neg] vs. SCH_Non_Neg [schizophrenia patients without predominant negative symptoms, SCH_Non_Neg], *p*< 0.05). Similar patterns were observed for CAP7 (k=7) and CAP3 (k=6) in the two-group comparison (*p* < 0.05). In the three-group analysis, k=8 and k=7 maintained consistent group differences (*p* < 0.05), while k=6 showed no significant differences (Fig. S9C).

**4.3  Comparison of CAP Clustering Sources**

We compared CAP results derived from three clustering sample sources: healthy controls (HC), schizophrenia patients (SCH), and all subjects combined (ALL). Spatial similarity analysis showed strong correlations (r=0.72–0.98 for matched pairs) between corresponding CAP states across these sources (Fig. S10B). Temporal dynamics analysis indicated that clustering based on HC data most effectively distinguished group differences (Fig. S10C-D), with significant effects observed in CAP3 dwell time, CAP3 occurrence rate, and CAP5 occurrence rate. In contrast, clustering based on SCH data yielded only a single significant difference (CAP4 occurrence rate), while clustering using all subjects did not reveal any significant group differences. These findings indicate that, while CAP spatial patterns are largely consistent across clustering sources, the choice of clustering sample can influence the sensitivity for detecting group differences in temporal dynamics. Using HC-derived CAPs provides a robust normative reference for evaluating disease-related alterations in brain dynamics (Yang et al., 2021).

**4.4 Parcellation Scheme Analysis**

To examine the robustness of CAP analysis across different parcellation schemes, we compared clustering performance using three established brain atlases: AAL-116, Brainnetome-246 (BN), and Schaefer-408. For each atlas, clustering metrics were calculated across k=2 to k=20, with identical preprocessing and CAP derivation procedures. All three parcellation schemes exhibited similar evaluation metric trends (Fig. S11A), with consistent inflection points around k=6~8. The Schaefer-408 atlas achieved higher clustering quality with Silhouette coefficient (0.087) compared to BN-246 (0.033) and AAL-116 (0.016), along with superior Calinski-Harabasz index (Schaefer: 23.39, BN: 18.67, AAL: 16.16). Spatial similarity analysis (Fig. S11C) revealed high correspondence between equivalent CAP states across atlases, with strongest spatial correlations between Schaefer-BN (max *r*=0.87), followed by AAL-BN (max *r*=0.83) and Schaefer-AAL (max *r*=0.73). The CAP temporal dynamics (DwellTime and OccurrenceRate) showed consistent group differences across atlases (Fig. S11D-E), with significant differences primarily observed between HC vs. SCH_Neg in matched CAP states (CAP3, CAP5, CAP7). This cross-atlas consistency was maintained in both two-group (HC vs. SCH) and three-group (HC vs. SCH_Neg vs. SCH_Non_Neg) comparisons, with similar effect directions and statistical significance patterns, supporting the robustness of CAP findings regardless of parcellation scheme choice.

**4.5 External Dataset Validation**

To assess the generalizability of our findings, we conducted a search for suitable external datasets, including COBRE (Center for Biomedical Research Excellence), UCLA (University of California, Los Angeles), and SRPBS (Strategic Research Program for Brain Sciences) databases. However, we found that patients with predominantly negative symptoms were limited in these datasets. For example, using our PANSS-based criteria, only 9 of 72 SCH patients in COBRE and 7 of 92 SCH patients in SRPBS met criteria for negative symptom predominance. Given these constraints, we focused on validating our general SCH vs HC findings using the COBRE dataset

For this validation analysis, we performed external validation using the COBRE dataset, applying the same quality control criteria as in the main sample. From the initial COBRE dataset of 146 subjects (74 HC, 72 SCH), we excluded one SCH subject with insufficient fMRI data (<150 timepoints), 3 HC and 12 SCH non-right-handed subjects, and subjects with excessive head motion (>2.5mm or >2.5 degrees; 3 HC and 10 SCH). After exclusion, 68 HC and 49 SCH subjects remained; for group comparisons, we used matched samples of 49 HC and 49 SCH (see Table S3 for demographic and clinical characteristics).

CAP analysis was conducted in two ways: (1) independent clustering using COBRE HC to derive CAP centers, and (2) applying CAP centers derived from our primary dataset, the Shanghai Mental Health Center (SMHC) dataset,  to COBRE data. Spatial similarity analysis revealed strong correspondence between matched CAP states across datasets (e.g., SMHC-CAP3↔COBRE-CAP3, *r*=0.92; SMHC-CAP7↔COBRE-CAP5, *r*=0.94), indicating robust spatial patterns (Fig. S12B-C). Temporal dynamics comparisons showed that key group differences were highly consistent (Fig. S12D): HC exhibited longer dwell times in CAP3 and higher occurrence rates in SMHC-CAP5 (corresponding to COBRE-CAP6) compared to SCH, while SCH showed higher occurrence rates in CAP3. The higher occurrence rates in SMHC-CAP7 for SCH were significant in both SMHC and independent COBRE clustering (as COBRE-CAP5), though this difference did not reach statistical significance when applying SMHC-derived centers to COBRE data. Overall, these results confirm that the main findings regarding altered brain state dynamics in schizophrenia are reproducible across independent datasets and analysis strategies.

**5. Medication Effects Sensitivity Analysis**

The potential confounding influence of antipsychotic medication on brain network dynamics was evaluated through a series of sensitivity analyses. For patients with available medication information, daily doses were standardized as chlorpromazine equivalents (CPZ). Three ANCOVA models were constructed for comparative analyses. The basic model included age, sex, and education as covariates, serving as our reference analysis. The duration model incorporated illness duration as an additional covariate, while the medication model included CPZ dosage for participants with available medication data. These parallel models allowed systematic assessment of how clinical variables might influence observed brain network differences.

In the two-group comparison of healthy controls and all schizophrenia patients, the inclusion of medication dosage or illness duration as covariates led to a reduction in the number of significant group differences in CAP features. As shown in Table S4, none of the features that were significant in the basic model remained significant after controlling for medication effects. When adjusting for illness duration, only a few features remained significant, including occurrence rate of CAP5 and CAP7, transition probability from CAP3 to CAP8, and several Markov trajectory entropy measures (CAP2 to CAP3, CAP4 to CAP3, and CAP5 to CAP3). These results indicate that both antipsychotic medication and illness duration may account for some of the observed group differences in CAP dynamics.

The three-group comparison among HC, SCH_Neg, and SCH_Non_Neg showed a similar pattern. As detailed in Table S5, only a limited number of the significant findings from the basic model persisted in the medication model, with just one comparison showing significance: the difference in iSAR for CAP2 between SCH_Neg and SCH_Non_Neg groups. In the duration model, more findings remained significant, including iSAR for CAP2, occurrence rate for CAP5, and transition probability from CAP5 to CAP6. Post-hoc pairwise comparisons confirmed that the difference in transition probability from CAP5 to CAP6 between SCH_Neg and SCH_Non_Neg remained robust after adjustment for illness duration (t=-2.756, *p*=0.007), though this finding was not significant in the medication model.


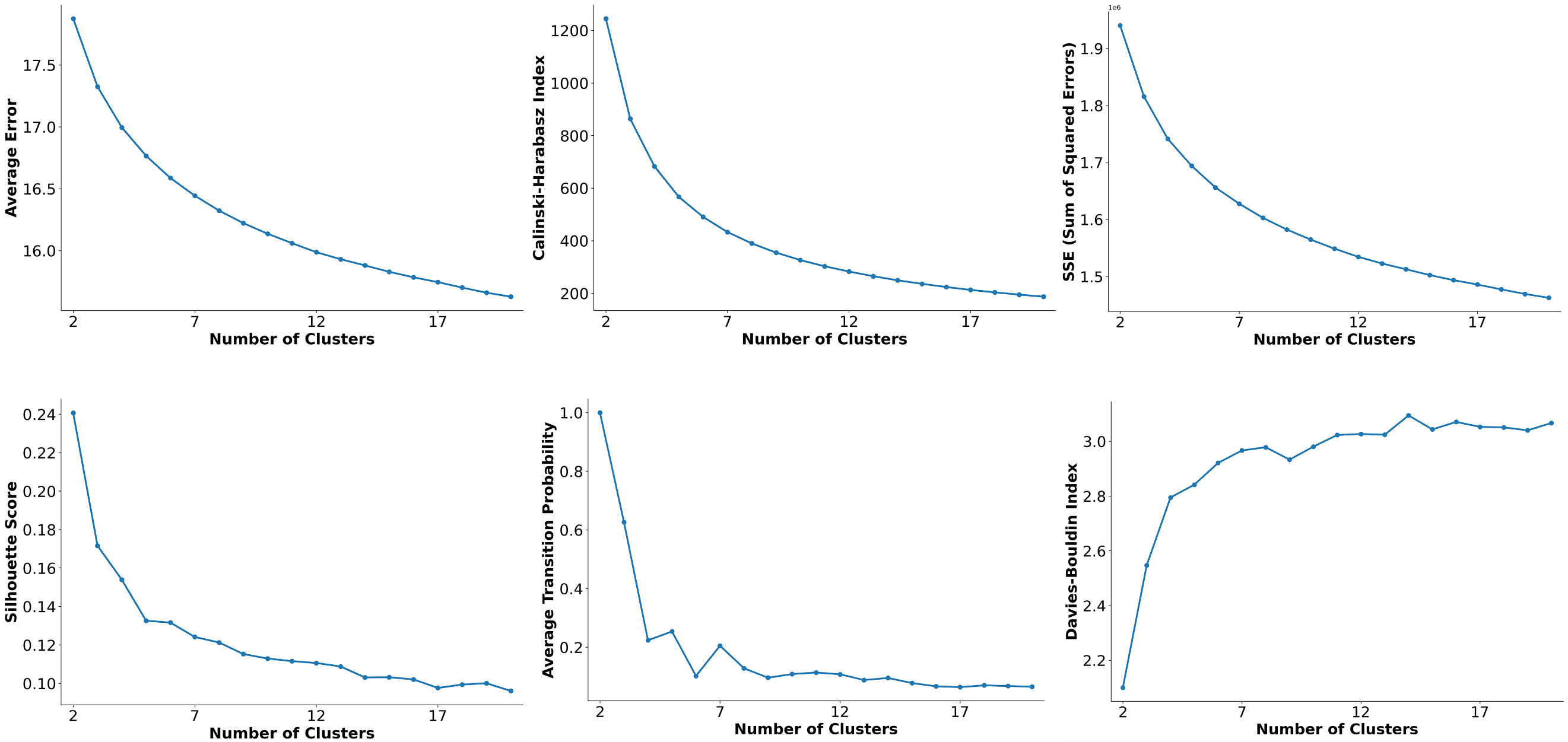


**Fig. S1** Evaluation metrics for different numbers of clusters

**
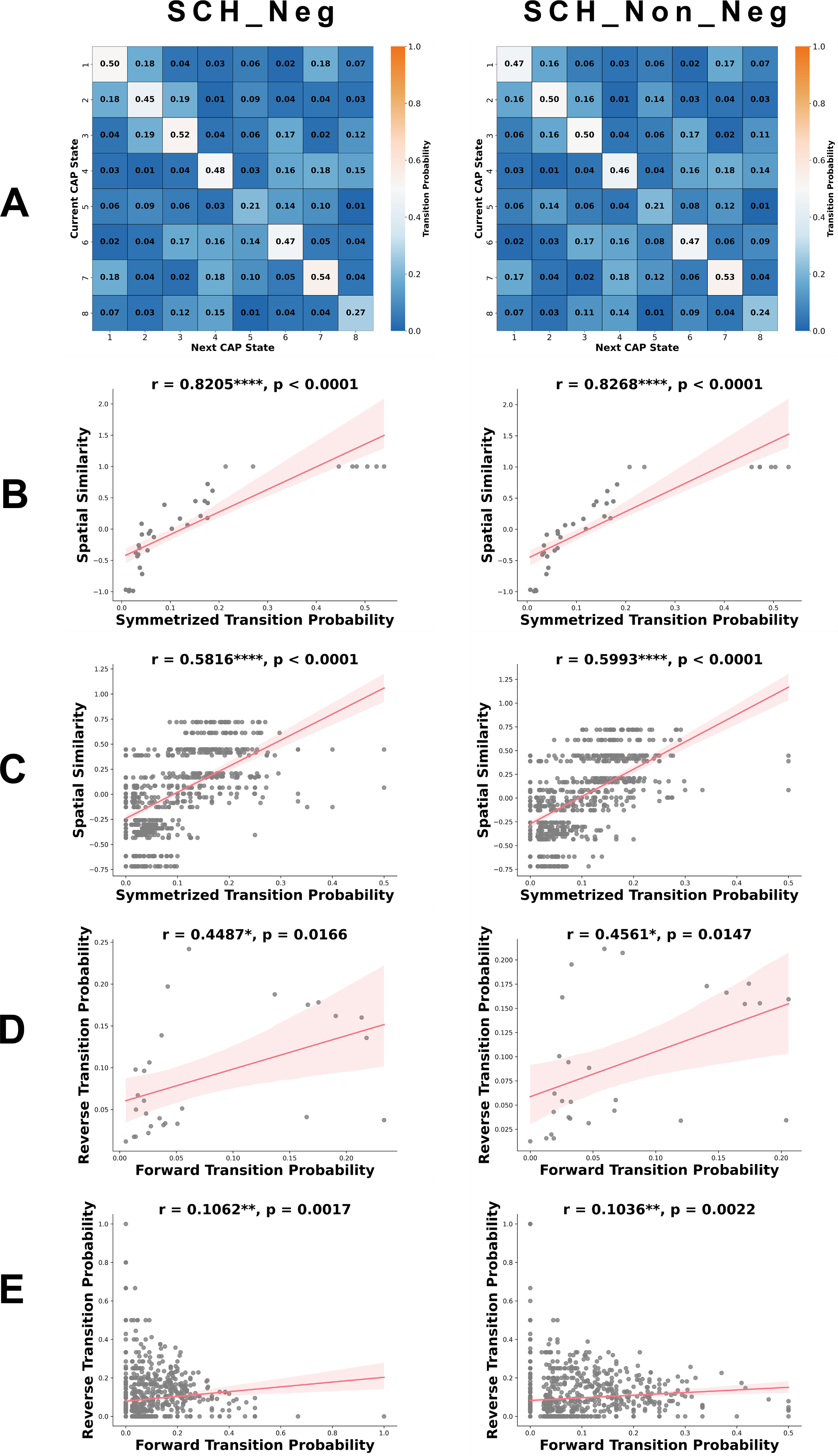
**

**Fig. S2.** Spatial similarity and transition dynamics of CAP states in SCH_Neg and SCH_Non_Neg groups. A) Group-level transition probability matrices for SCH_Neg and SCH_Non_Neg groups. B) Correlations between spatial similarity and symmetrized transition probabilities at the group level for SCH_Neg and SCH_Non_Neg groups. C) Correlations between spatial similarity and symmetrized transition probabilities at the individual level, excluding anti-correlated CAP pairs, for SCH_Neg and SCH_Non_Neg groups. D) Transition probability symmetry analyses at the group level for SCH_Neg and SCH_Non_Neg groups. E) Transition probability symmetry analyses at the individual level for SCH_Neg and SCH_Non_Neg groups. For B-E, each point represents a CAP state pair, excluding diagonal elements (within-state transitions). Shaded areas indicate 95% confidence intervals. Significance levels*:***p*< 0.05, ***p* < 0.01, ****p*< 0.001, *****p* < 0.0001.


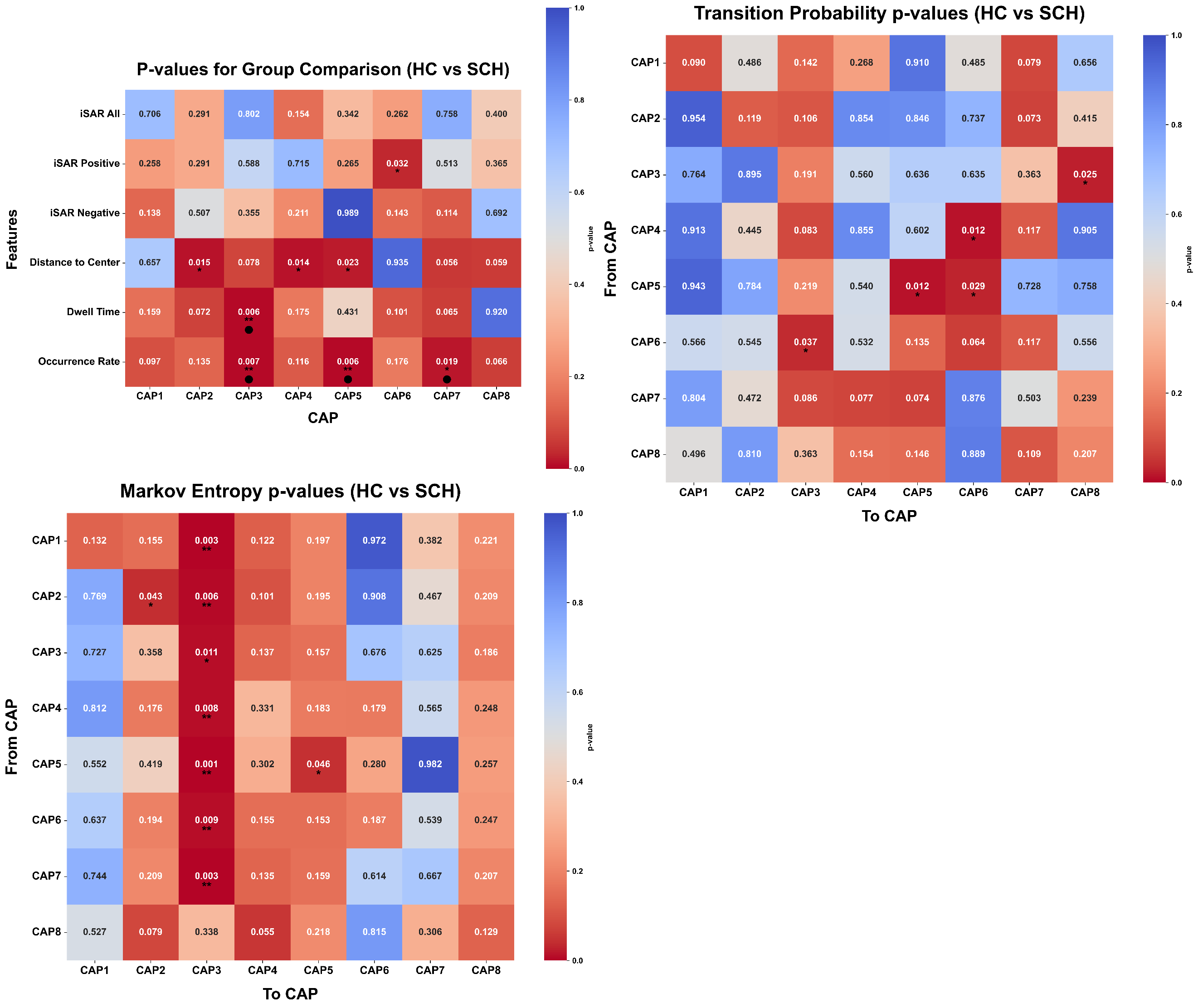


**Fig. S3.** Statistical comparison of CAP features between healthy controls (HC) and the whole schizophrenia patients (SCH). Values represent p-values from group comparisons, with significance levels indicated by asterisks: * *p <* 0.05*, *** *p* < 0.01,***** *p* < 0.001. Black dots indicate significance after FDR correction for multiple comparisons. Cooler colors (blue) indicate higher p-values, while warmer colors (red) indicate lower p-values.


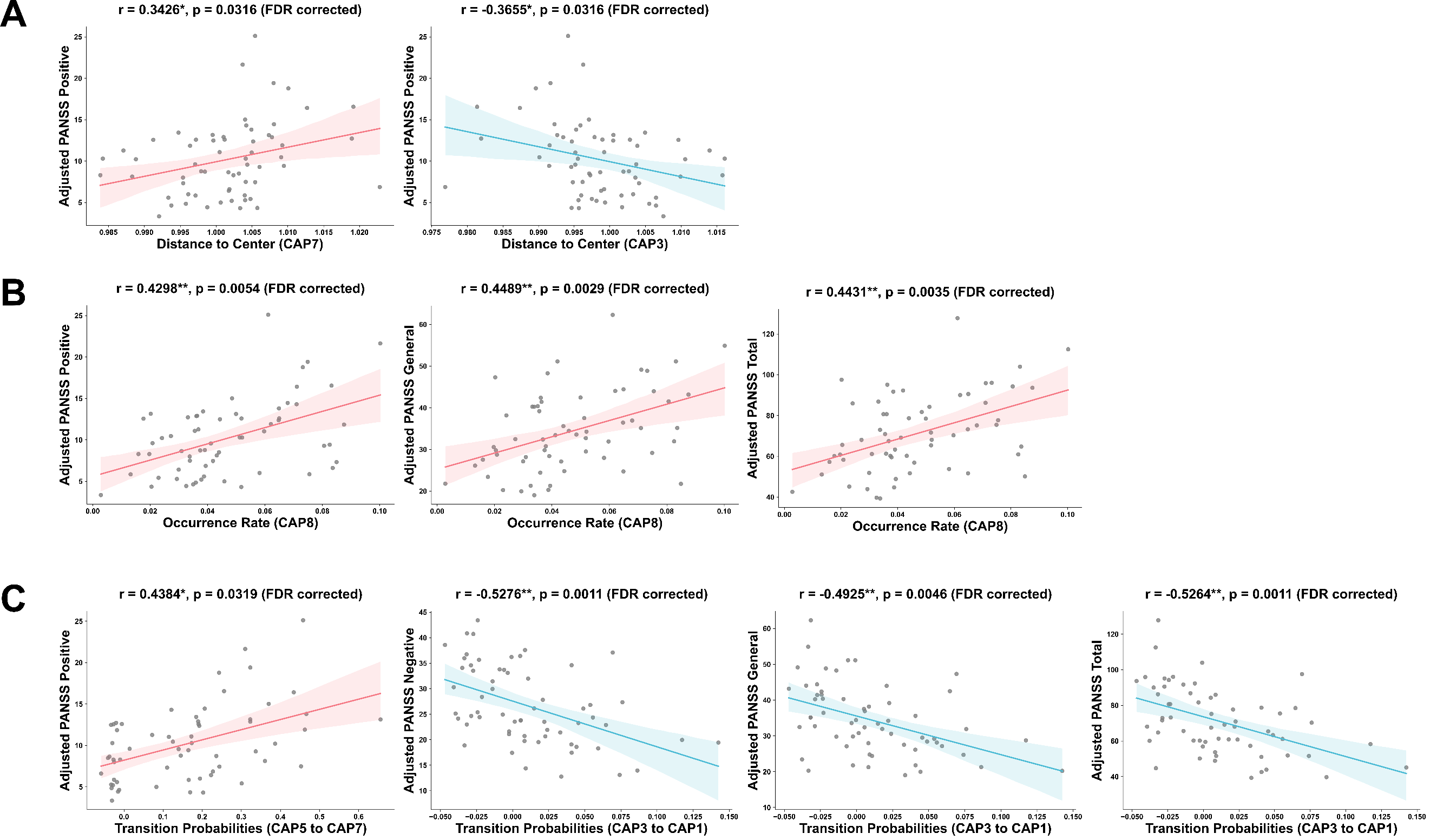


**Fig. S4.** Significant correlations of CAP features and clinical symptoms in the whole schizophrenia group.  Only correlations that remained significant after FDR correction are shown. FDR correction was applied separately for each clinical scale within each CAP feature. (A) Distance to center with PANSS scores. (B) Occurrence rate with PANSS scores. (C) Transition probabilities with PANSS. Red and blue lines indicate positive and negative correlations, respectively. Gray dots represent individual data points. Significant levels:**p* < 0.05,** *p* < 0.01 (FDR corrected).

**
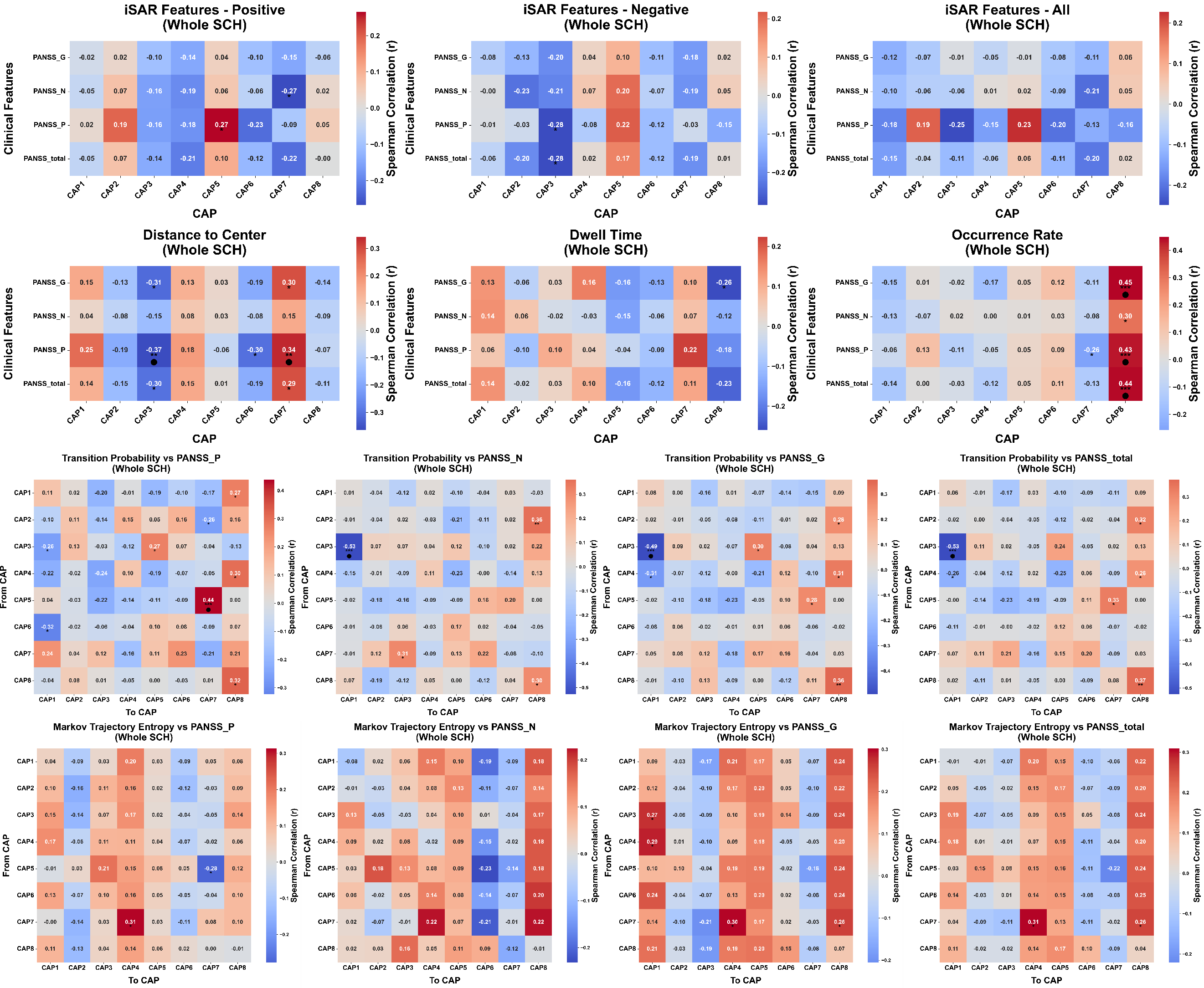
Fig. S5.** Heatmaps display Spearman correlation coefficients (r) between CAP features and PANSS scores in whole schizophrenia patients. Color intensity indicates correlation strength and direction (red = positive, blue = negative). Statistical significance: * *p* < 0.05, ** *p* < 0.01, *** *p* < 0.001; black dots indicate significance after FDR correction (*p* < 0.05). PANSS: Positive and Negative Syndrome Scale; P: Positive symptoms; N: Negative symptoms; G: General psychopathology; CAP: Co-Activation Pattern.

**
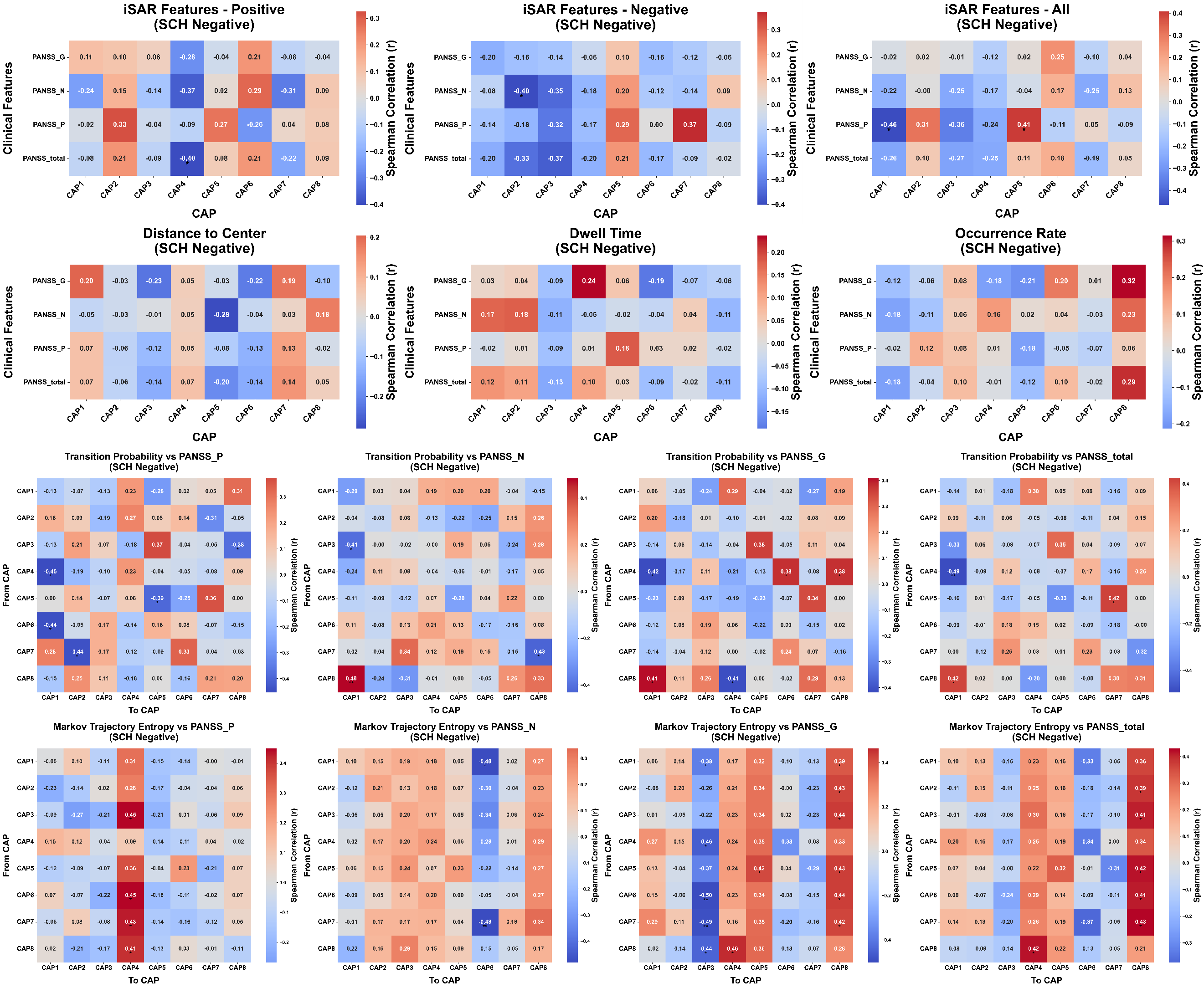
Fig. S6.** Heatmaps display Spearman correlation coefficients (r) between CAP features and PANSS scores in schizophrenia patients with predominantly negative symptoms. Color intensity indicates correlation strength and direction (red = positive, blue = negative). Statistical significance: * *p* < 0.05, ** *p* < 0.01, *** *p* < 0.001; black dots indicate significance after FDR correction (*p* < 0.05). PANSS: Positive and Negative Syndrome Scale; P: Positive symptoms; N: Negative symptoms; G: General psychopathology; CAP: Co-Activation Pattern.


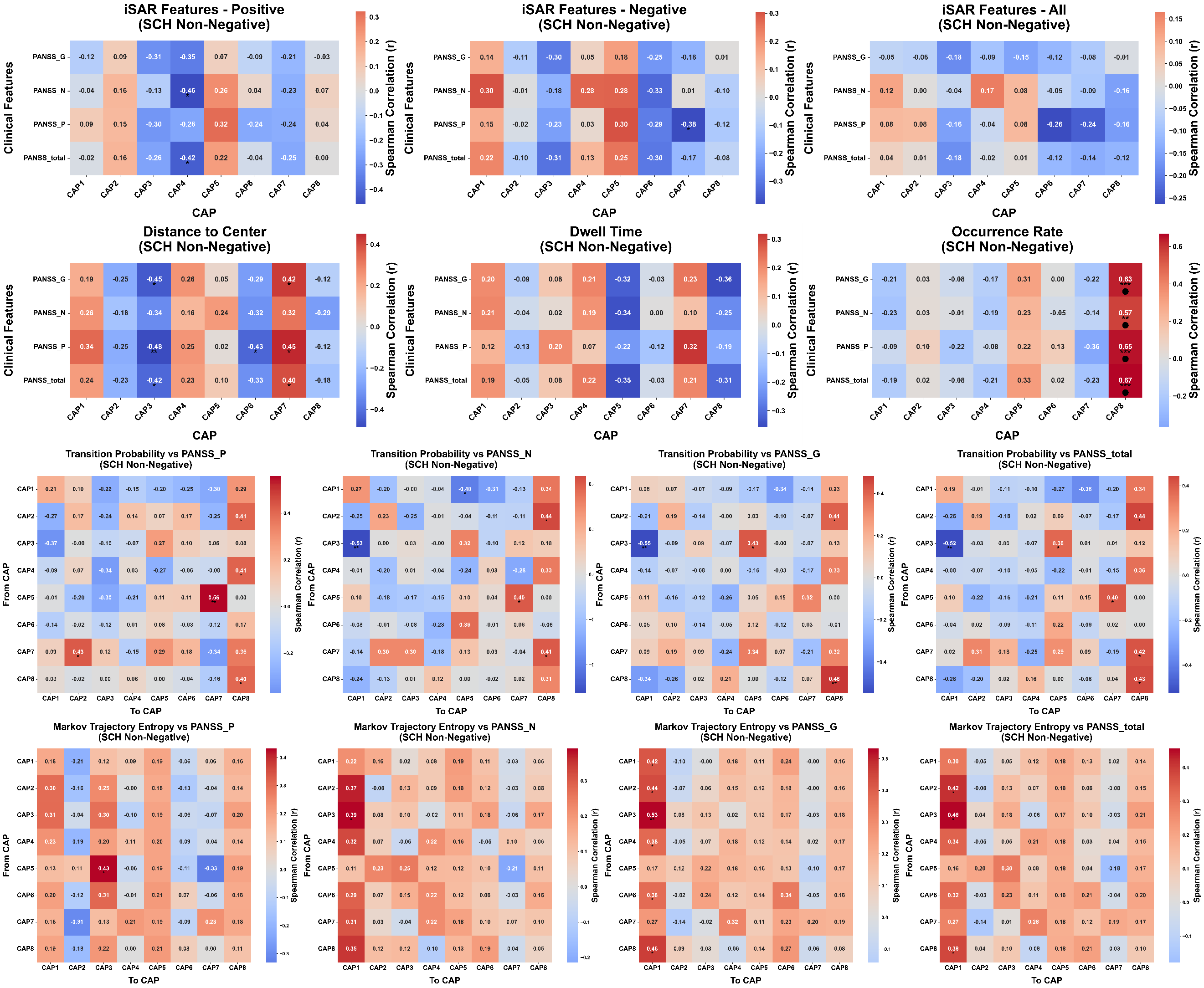


**Fig. S7.** Heatmaps display Spearman correlation coefficients (r) between CAP features and PANSS scores in schizophrenia patients with non-predominant negative symptoms. Color intensity indicates correlation strength and direction (red = positive, blue = negative). Statistical significance: * *p* < 0.05, ** *p* < 0.01, *** *p* < 0.001; black dots indicate significance after FDR correction (*p* < 0.05). PANSS: Positive and Negative Syndrome Scale; P: Positive symptoms; N: Negative symptoms; G: General psychopathology; CAP: Co-Activation Pattern.


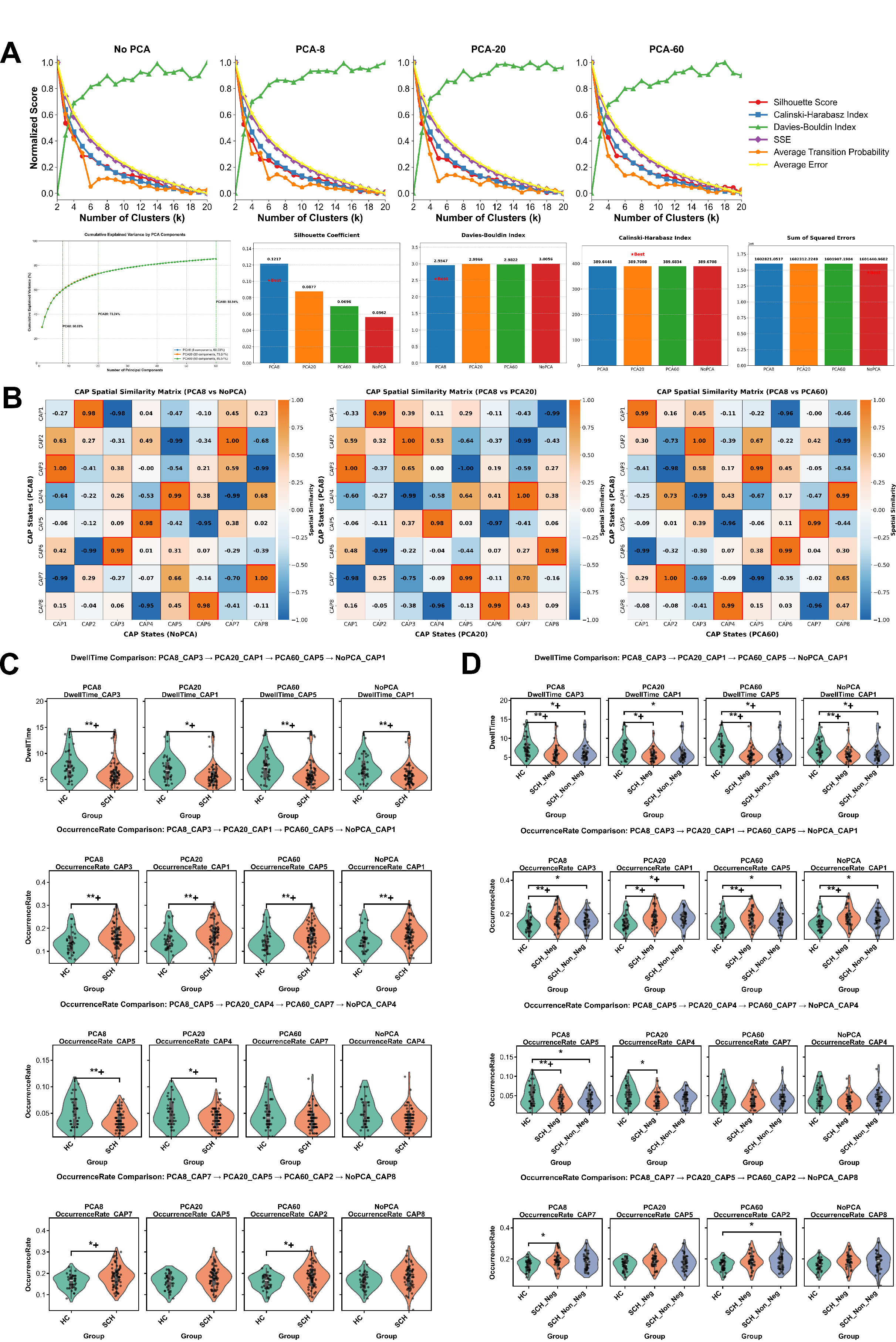


**Figure S8.** Reproducibility analysis of CAP states and their dynamic properties across different PCA conditions**.** (A) Clustering quality evaluation across different PCA conditions. (B) Spatial similarity matrices comparing CAP states between PCA conditions. (C) Comparison of temporal dynamics between healthy controls (HC) and the whole schizophrenia patients aross PCA conditions. (D) Subgroup comparison (HC vs SCH_Neg, HC vs SCH_Non_Neg, SCH_Neg vs SCH_Non_Neg) of temporal dynamics cross PCA conditions. Significance : * *p* < 0.05*, ** p* < 0.01, *** *p* <0.001, + significant after FDR correction*.*


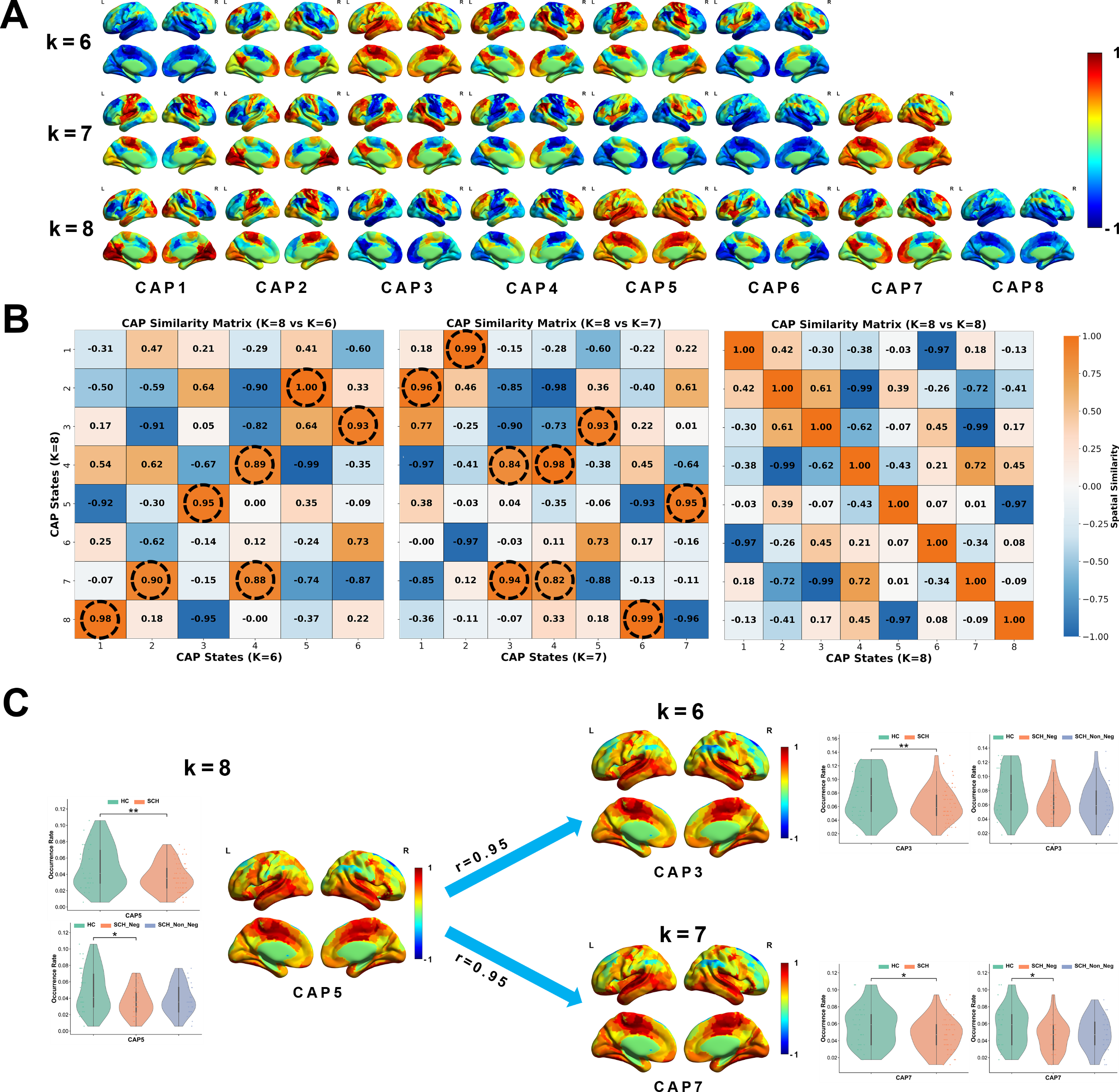


**Fig. S9.** Reproducibility analysis of CAP states and their dynamic properties across different cluster numbers. (A) Spatial CAP maps of k=6, k=7 and k=8. (B) Spatial similarity matrix showing correlations between CAPs of k=8 and those of k=6, k=7, and k=8 itself. (C) Consistency of group differences in occurrence rate across different k values. The occurrence rate of CAP5 (k=8) showed consistent group differences in both two-group (HC vs. SCH) and three-group (HC vs. SCH_Neg vs. SCH_Non_Neg) comparisons. Similar patterns were observed for CAP7 (k=7) and CAP3 (k=6) in the two-group comparison. In the three-group comparison, k=8 and k=7 maintained consistency, while k=6 showed no significant differences. Significance levels: **p* < 0.05, ***p* < 0.01.


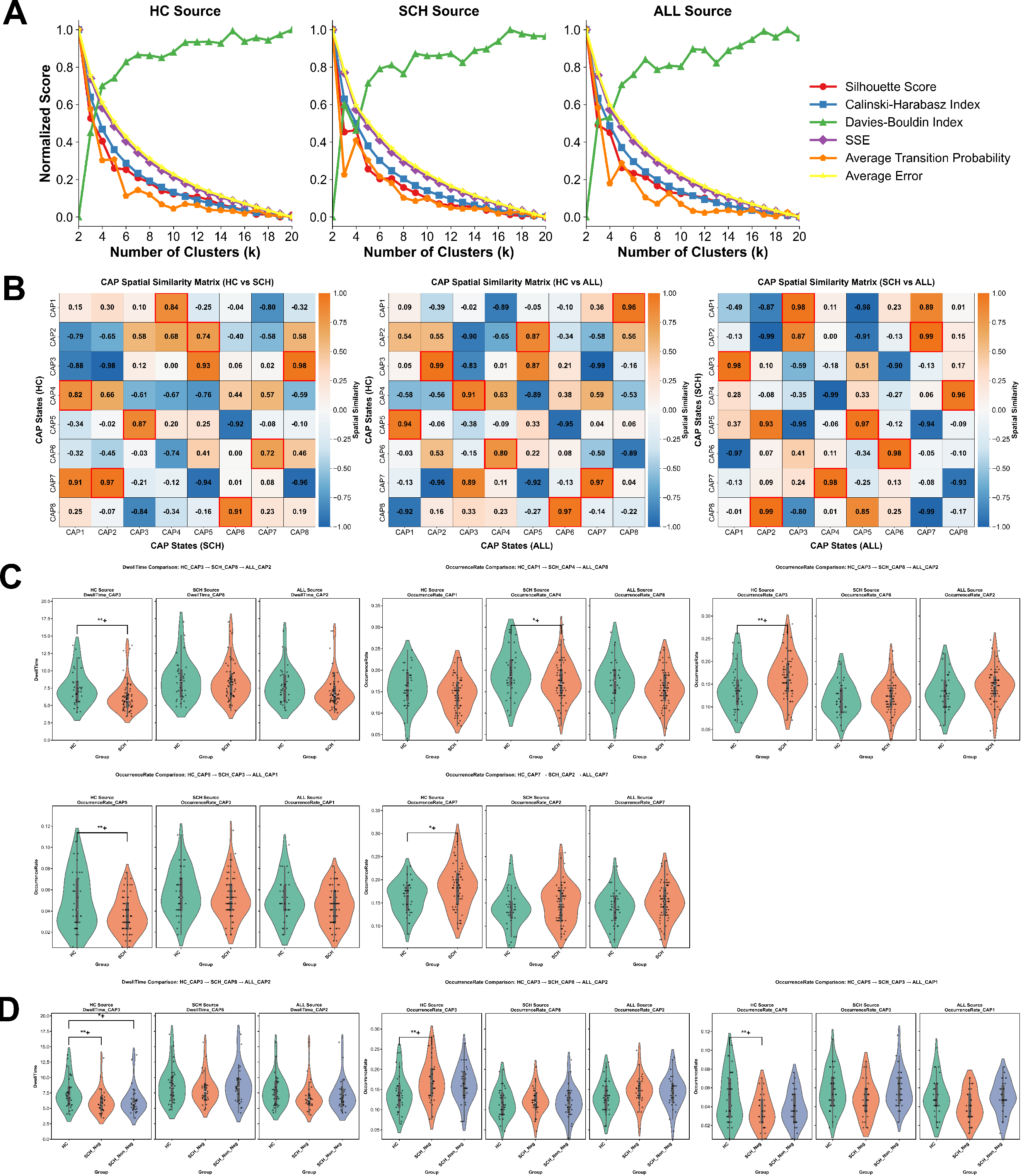


**Fig. S10.** Validation of CAP clustering sources. (A) Clustering quality evaluation across different clustering sources. (B) Spatial similarity matrices between CAP states derived from different sources (HC vs. ALL, HC vs. SCH, SCH vs. ALL).  (C) Comparison of temporal dynamics between healthy controls (HC) and the whole schizophrenia patients across different clustering sources. (D) Subgroup comparison (HC vs SCH_Neg, HC vs SCH_Non_Neg, SCH_Neg vs SCH_Non_Neg) of temporal dynamics across different clustering sources. Significance : * *p* < 0.05*, ** p* < 0.01, *** *p* <0.001, + significant after FDR correction*.*

**
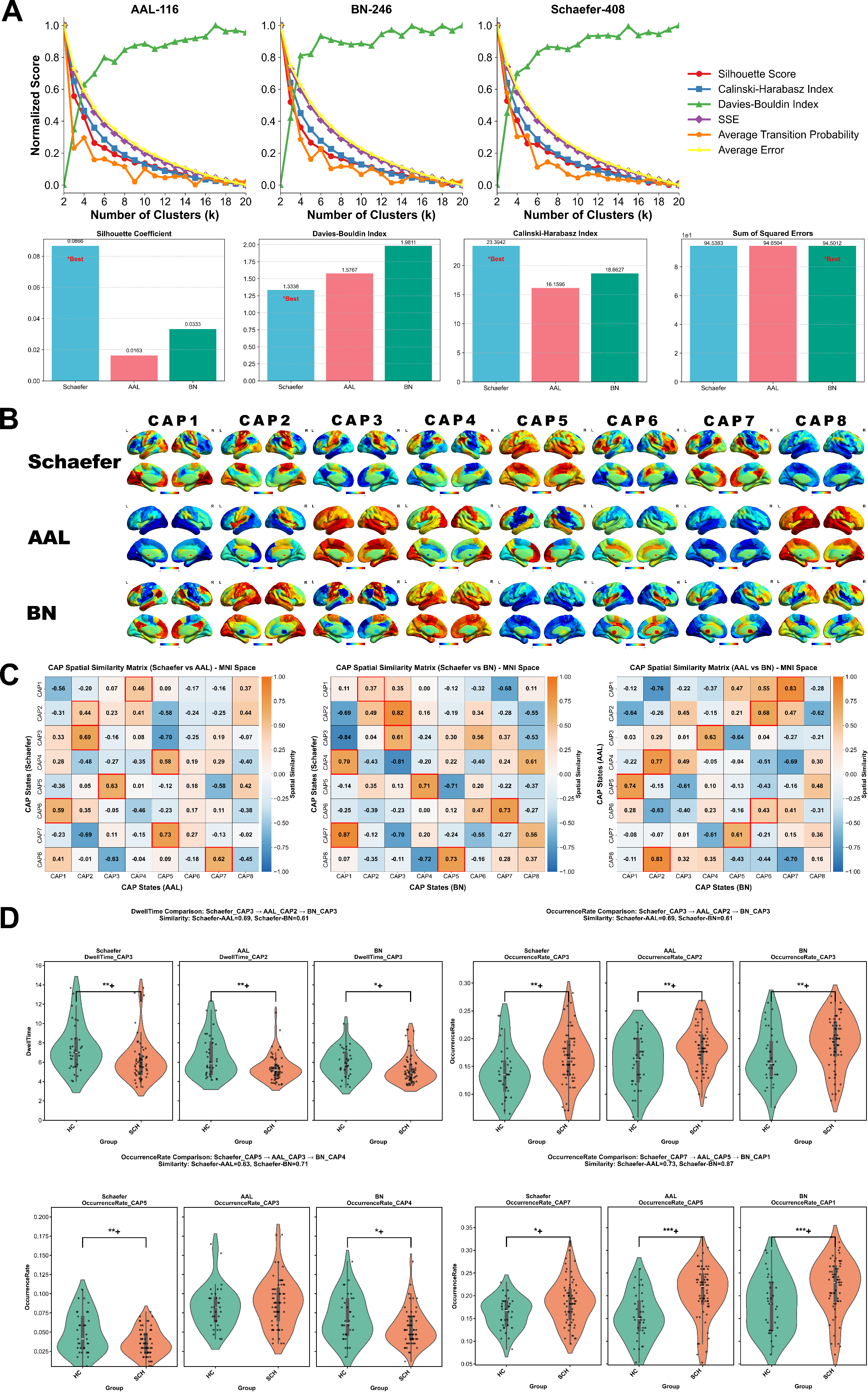
**

**
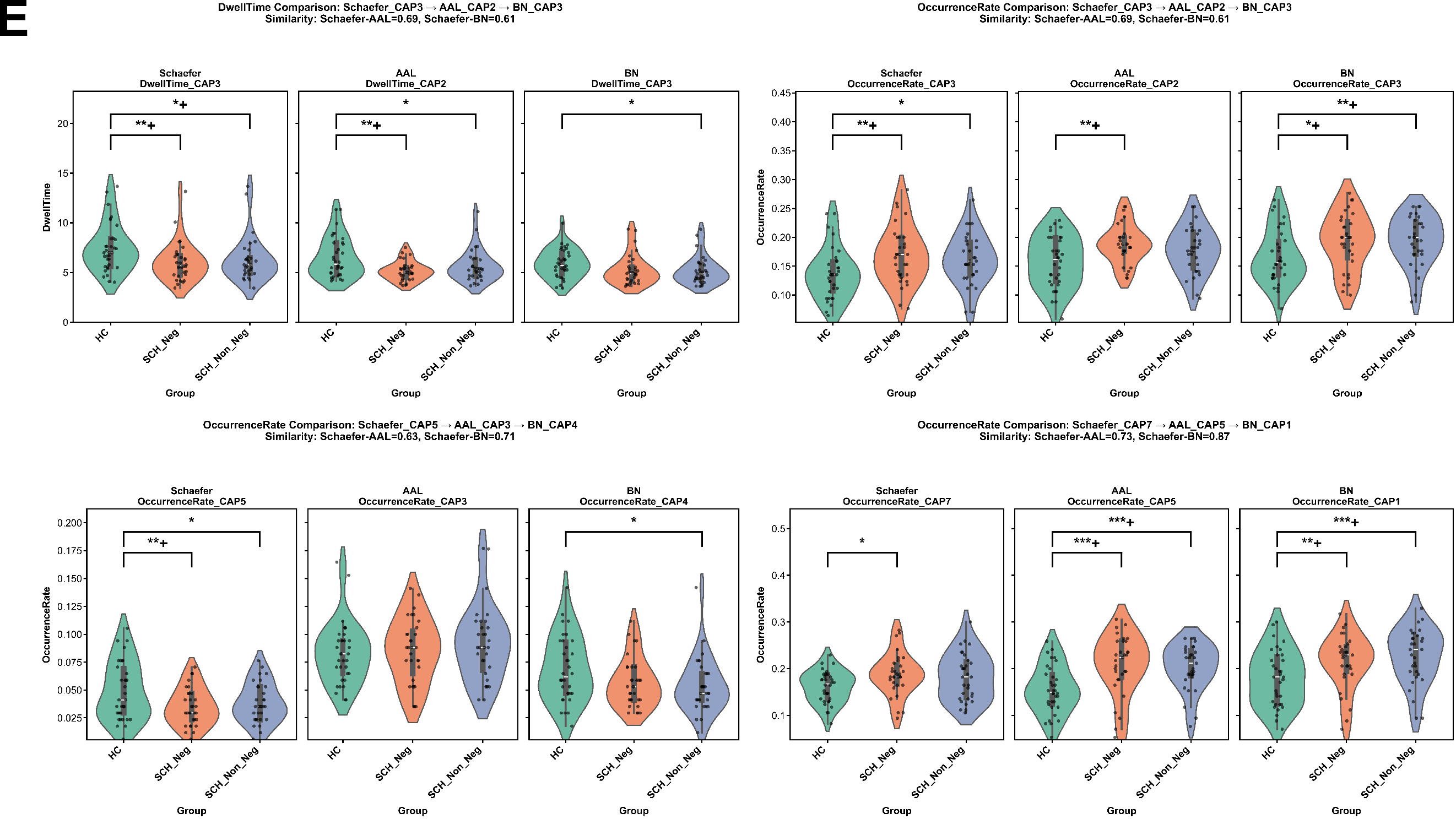
Fig. S11.** Parcellation scheme analysis. (A) Clustering quality evaluation across different atlases. (B) Spatial CAP maps arcoss different atlases. (C) Spatial similarity matrices between CAP states derived from different atlases. (D) Comparison of temporal dynamics between healthy controls (HC) and the whole schizophrenia patients across different atlases. (E) Subgroup comparison (HC vs SCH_Neg, HC vs SCH_Non_Neg, SCH_Neg vs SCH_Non_Neg) of temporal dynamics across different atlases. Significance : * *p* < 0.05*, ** p* < 0.01, *** *p* <0.001, + significant after FDR correction*.*

**
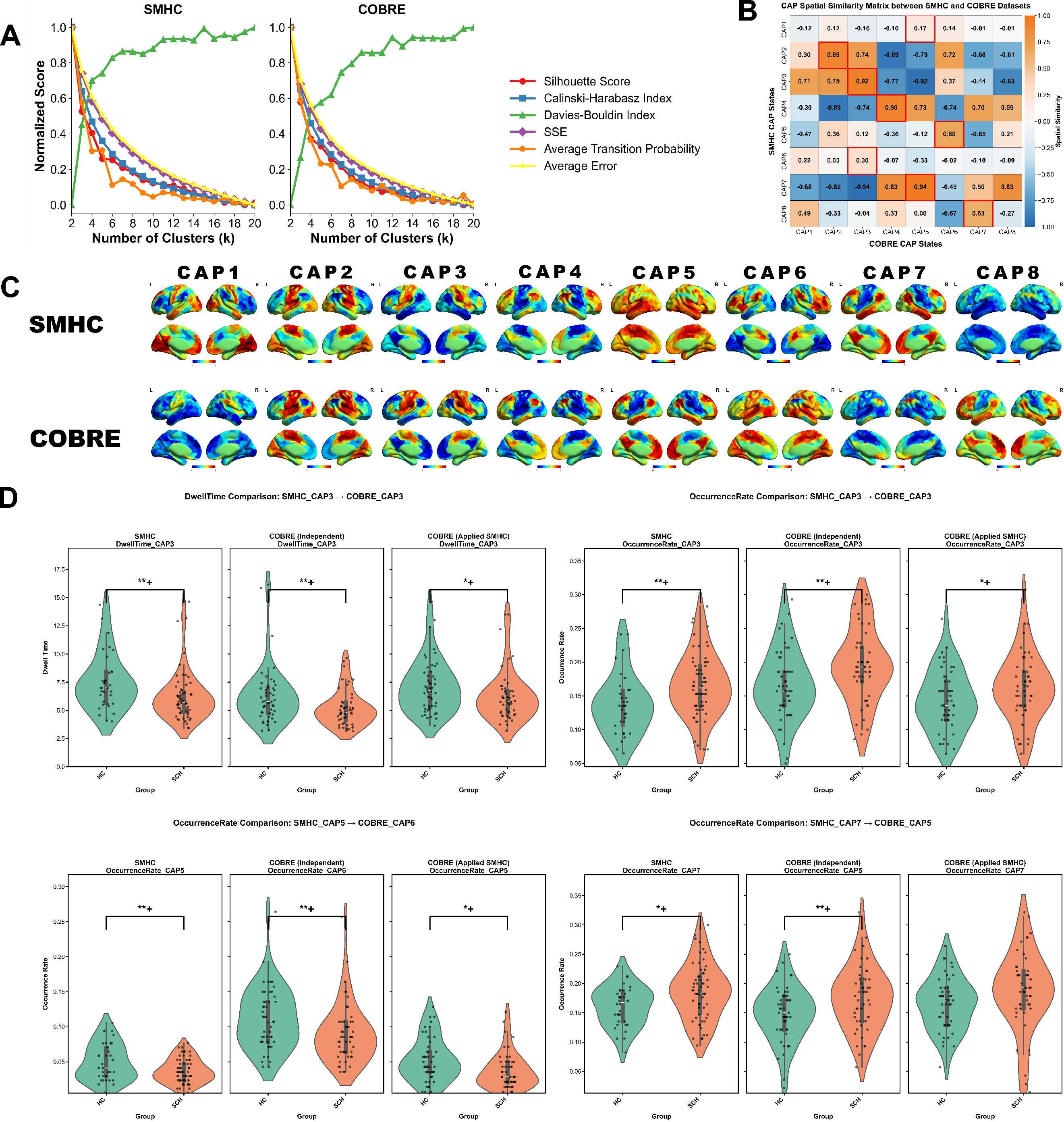
Fig. S12**. External dataset validation using COBRE data. (A) Clustering quality evaluation of SMHC data and COBRE data. (B) Spatial similarity matrix between SMHC and COBRE CAP states. (C) Spatial CAP maps of SMHC data and COBRE data. (D) Comparison of temporal dynamics across different datasets. Significance : * *p* < 0.05, ** *p* < 0.01, *** *p* <0.001, + significant after FDR correction.

**Table S1.** Significant ANCOVA results for spatial stability and network dynamics measures differences between HC and the whole SCH groups. The configuration was k = 8 CAP states.

| **Feature** | **CAP** | **HC (Mean ± SD)** | **SCH (Mean ± SD)** | **F value** | **P value** |
| --- | --- | --- | --- | --- | --- |
| iSAR (positive) | CAP6 | 0.612 ± 0.033 | 0.626 ± 0.029 | 4.75 | 0.032* |
| Distance to Center | CAP2 | 1.000 ± 0.005 | 1.002 ± 0.007 | 6.10 | 0.015* |
| Distance to Center | CAP4 | 1.000 ± 0.006 | 0.998 ± 0.007 | 6.30 | 0.014* |
| Distance to Center | CAP5 | 1.000 ± 0.004 | 1.001 ± 0.003 | 5.32 | 0.023* |
| Dwell Time | CAP3 | 7.468 ± 2.413 | 6.166 ± 2.095 | 8.01 | 0.006**+ |
| Occurrence Rate | CAP3 | 0.137 ± 0.044 | 0.165 ± 0.047 | 7.50 | 0.007**+ |
| Occurrence Rate | CAP5 | 0.049 ± 0.026 | 0.037 ± 0.017 | 7.95 | 0.006**+ |
| Occurrence Rate | CAP7 | 0.161 ± 0.034 | 0.185 ± 0.047 | 5.73 | 0.019*+ |
| Transition Probability | CAP3 to CAP8 | 0.064 ± 0.061 | 0.038 ± 0.040 | 5.19 | 0.025* |
| Transition Probability | CAP4 to CAP6 | 0.177 ± 0.079 | 0.139 ± 0.057 | 6.57 | 0.012* |
| Transition Probability | CAP5 to CAP5 | 0.292 ± 0.192 | 0.211 ± 0.168 | 6.58 | 0.012* |
| Transition Probability | CAP5 to CAP6 | 0.104 ± 0.119 | 0.177 ± 0.191 | 4.93 | 0.029* |
| Transition Probability | CAP6 to CAP3 | 0.140 ± 0.079 | 0.175 ± 0.090 | 4.50 | 0.037* |
| Markov Trajectory Entropy | CAP1 to CAP3 | 28.325 ± 6.885 | 24.276 ± 6.056 | 9.47 | 0.003** |
| Markov Trajectory Entropy | CAP2 to CAP2 | 13.534 ± 3.581 | 15.430 ± 4.976 | 4.22 | 0.043* |
| Markov Trajectory Entropy | CAP2 to CAP3 | 23.367 ± 7.366 | 19.442 ± 5.896 | 7.93 | 0.006** |
| Markov Trajectory Entropy | CAP3 to CAP3 | 15.181 ± 4.794 | 12.650 ± 4.184 | 6.69 | 0.011* |
| Markov Trajectory Entropy | CAP4 to CAP3 | 28.926 ± 7.699 | 25.015 ± 6.504 | 7.31 | 0.008** |
| Markov Trajectory Entropy | CAP5 to CAP3 | 28.189 ± 8.152 | 22.854 ± 7.388 | 10.90 | 0.001** |
| Markov Trajectory Entropy | CAP5 to CAP5 | 54.784 ± 42.286 | 72.553 ± 51.638 | 4.09 | 0.046* |
| Markov Trajectory Entropy | CAP6 to CAP3 | 24.759 ± 8.418 | 20.520 ± 6.896 | 7.23 | 0.009** |
| Markov Trajectory Entropy | CAP7 to CAP3 | 30.413 ± 7.475 | 26.384 ± 5.792 | 9.22 | 0.003** |

**Notes:** The analysis was performed using ANCOVA with age, sex, and education as covariates. Significance levels: * *p* < 0.05, ** *p* < 0.01, *** *p* <0.001, + significant after FDR correction (*p* <0.05). FDR correction was applied within each feature family.

**Table S2.** Post-hoc pairwise comparison results for significant ANCOVA group effects on spatial stability and network dynamics measures among HC, SCH_Neg, and SCH_Non_Neg groups. The configuration was k = 8 CAP states.

| **Feature** | **CAP** | **Comparison** | **Group A Mean** | **Group B Mean** | **T value** | **P value** |
| --- | --- | --- | --- | --- | --- | --- |
| iSAR | CAP2 | HC vs SCH_Neg | 0.766 | 0.751 | -2.16 | 0.034* |
| iSAR | CAP2 | SCH_Neg vs SCH_Non_Neg | 0.751 | 0.765 | 2.36 | 0.020* |
| Distance to Center | CAP4 | HC vs SCH_Neg | 1.000 | 0.997 | -2.36 | 0.021* |
| Distance to Center | CAP4 | HC vs SCH_Non_Neg | 1.000 | 0.998 | -2.00 | 0.048* |
| Dwell Time | CAP3 | HC vs SCH_Neg | 7.468 | 6.045 | -2.70 | 0.008**+ |
| Dwell Time | CAP3 | HC vs SCH_Non_Neg | 7.468 | 6.287 | -2.22 | 0.029*+ |
| Occurrence Rate | CAP3 | HC vs SCH_Neg | 0.137 | 0.169 | 2.67 | 0.009**+ |
| Occurrence Rate | CAP3 | HC vs SCH_Non_Neg | 0.137 | 0.162 | 2.10 | 0.038* |
| Occurrence Rate | CAP5 | HC vs SCH_Neg | 0.049 | 0.035 | -2.77 | 0.007**+ |
| Occurrence Rate | CAP5 | HC vs SCH_Non_Neg | 0.049 | 0.039 | -2.15 | 0.034* |
| Transition Probability | CAP1 to CAP3 | HC vs SCH_Non_Neg | 0.041 | 0.068 | 2.46 | 0.016*+ |
| Transition Probability | CAP1 to CAP3 | SCH_Neg vs SCH_Non_Neg | 0.040 | 0.068 | 2.37 | 0.020*+ |
| Transition Probability | CAP4 to CAP6 | HC vs SCH_Neg | 0.177 | 0.137 | -2.27 | 0.026*+ |
| Transition Probability | CAP4 to CAP6 | HC vs SCH_Non_Neg | 0.177 | 0.141 | -2.17 | 0.033*+ |
| Transition Probability | CAP5 to CAP5 | HC vs SCH_Neg | 0.292 | 0.214 | -2.18 | 0.032*+ |
| Transition Probability | CAP5 to CAP5 | HC vs SCH_Non_Neg | 0.292 | 0.208 | -2.25 | 0.027*+ |
| Transition Probability | CAP5 to CAP6 | HC vs SCH_Neg | 0.104 | 0.233 | 3.45 | 0.001***+ |
| Transition Probability | CAP5 to CAP6 | SCH_Neg vs SCH_Non_Neg | 0.233 | 0.120 | -2.84 | 0.006**+ |
| Transition Probability | CAP8 to CAP6 | SCH_Neg vs SCH_Non_Neg | 0.061 | 0.163 | 2.51 | 0.014*+ |
| Markov Trajectory Entropy | CAP1 to CAP3 | HC vs SCH_Neg | 28.325 | 24.136 | -2.72 | 0.008**+ |
| Markov Trajectory Entropy | CAP1 to CAP3 | HC vs SCH_Non_Neg | 28.325 | 24.416 | -2.6 | 0.011*+ |
| Markov Trajectory Entropy | CAP2 to CAP3 | HC vs SCH_Neg | 23.367 | 18.437 | -3.04 | 0.003**+ |
| Markov Trajectory Entropy | CAP3 to CAP3 | HC vs SCH_Neg | 15.181 | 12.125 | -2.72 | 0.008**+ |
| Markov Trajectory Entropy | CAP4 to CAP3 | HC vs SCH_Neg | 28.926 | 24.914 | -2.4 | 0.019*+ |
| Markov Trajectory Entropy | CAP4 to CAP3 | HC vs SCH_Non_Neg | 28.926 | 25.115 | -2.28 | 0.025*+ |
| Markov Trajectory Entropy | CAP5 to CAP3 | HC vs SCH_Neg | 28.189 | 22.186 | -3.21 | 0.002**+ |
| Markov Trajectory Entropy | CAP5 to CAP3 | HC vs SCH_Non_Neg | 28.189 | 23.522 | -2.55 | 0.013*+ |
| Markov Trajectory Entropy | CAP6 to CAP3 | HC vs SCH_Neg | 24.759 | 19.983 | -2.62 | 0.010*+ |
| Markov Trajectory Entropy | CAP6 to CAP3 | HC vs SCH_Non_Neg | 24.759 | 21.056 | -2.06 | 0.042* |
| Markov Trajectory Entropy | CAP7 to CAP3 | HC vs SCH_Neg | 30.413 | 25.802 | -3.0 | 0.003**+ |
| Markov Trajectory Entropy | CAP7 to CAP3 | HC vs SCH_Non_Neg | 30.413 | 26.967 | -2.3 | 0.024*+ |

**Notes:** Significance levels: * *p* < 0.05, ** *p* < 0.01, *** *p* < 0.001, + significant after FDR correction within each feature comparison *(p* < 0.05). FDR correction was applied within each feature's post-hoc comparisons separately.

**Table S3. Demographic and Clinical Characteristics of the COBRE Dataset**

| **Characteristic** | **HC (n = 68)** | **HC (n = 49)** | **SZ (n = 49)** | **P value** |
| --- | --- | --- | --- | --- |
| Age (years) | 35.68 ± 11.07 | 37.29 ± 12.03 | 38.51 ± 14.13 | 0.645 |
| Sex (Male / Female) | 46 / 22 | 38/11 | 38/11 |  |
| Education (years) | 13.90 ± 1.74 | 14.07 ± 1.72 | 13.07 ± 1.76 | 0.009** |
| PANSS positive | - | - | 14.24 ± 4.57 | - |
| PANSS negative | - | - | 14.61 ± 5.26 | - |
| PANSS general | - | - | 32.10 ± 8.53 | - |
| PANSS total | - | - | 60.96 ± 13.92 | - |

**Note:** HC = Healthy Controls; SZ = Schizophrenia patients; PANSS = Positive and Negative Syndrome Scale. * *p* < 0.05, ** *p* < 0.01.

**Table S4. Sensitivity analysis of CAP features in the two-group (HC vs the whole SCH) comparison under medication and duration models.**

| **Feature** | **CAP** | **Medication Model_**  **F-value** | **Medication Model_**  **p-value** | **Medication Model_**  **Effect Size** | **Duration Model_**  **F-value** | **Duration Model_**  **p-value** | **Duration Model_**  **Effect Size** |
| --- | --- | --- | --- | --- | --- | --- | --- |
| iSAR (positive) | CAP6 | 1.886 | 0.173 | 0.022 | 3.472 | 0.066 | 0.037 |
| Distance to Center | CAP2 | 0.829 | 0.365 | 0.010 | 1.948 | 0.166 | 0.021 |
| Distance to Center | CAP4 | 0.921 | 0.340 | 0.011 | 2.107 | 0.150 | 0.023 |
| Distance to Center | CAP5 | 0.365 | 0.548 | 0.004 | 2.335 | 0.130 | 0.026 |
| Dwell Time | CAP3 | 1.613 | 0.208 | 0.019 | 2.878 | 0.093 | 0.031 |
| Occurrence Rate | CAP3 | 0.958 | 0.331 | 0.011 | 3.918 | 0.051 | 0.042 |
| Occurrence Rate | CAP5 | 1.102 | 0.297 | 0.013 | 6.077 | **0.016*+** | 0.064 |
| Occurrence Rate | CAP7 | 3.468 | 0.066 | 0.041 | 4.010 | **0.048*** | 0.043 |
| Transition Probability | CAP3 to CAP8 | 1.571 | 0.214 | 0.019 | 4.084 | **0.046*** | 0.044 |
| Transition Probability | CAP4 to CAP6 | 1.120 | 0.293 | 0.013 | 3.199 | 0.077 | 0.035 |
| Transition Probability | CAP5 to CAP5 | 0.031 | 0.860 | 0 | 1.739 | 0.191 | 0.019 |
| Transition Probability | CAP5 to CAP6 | 0.505 | 0.479 | 0.006 | 0.995 | 0.321 | 0.011 |
| Transition Probability | CAP6 to CAP3 | 1.305 | 0.257 | 0.016 | 3.003 | 0.087 | 0.033 |
| Markov Trajectory Entropy | CAP1 to CAP3 | 0.992 | 0.322 | 0.012 | 3.929 | 0.051 | 0.042 |
| Markov Trajectory Entropy | CAP2 to CAP2 | 0.904 | 0.344 | 0.011 | 2.261 | 0.136 | 0.025 |
| Markov Trajectory Entropy | CAP2 to CAP3 | 0.209 | 0.649 | 0.003 | 4.230 | **0.043*** | 0.045 |
| Markov Trajectory Entropy | CAP3 to CAP3 | 0.769 | 0.383 | 0.009 | 2.544 | 0.114 | 0.028 |
| Markov Trajectory Entropy | CAP4 to CAP3 | 1.298 | 0.258 | 0.016 | 4.517 | **0.036*** | 0.048 |
| Markov Trajectory Entropy | CAP5 to CAP3 | 2.497 | 0.118 | 0.029 | 4.969 | **0.028*** | 0.053 |
| Markov Trajectory Entropy | CAP5 to CAP5 | 1.366 | 0.246 | 0.016 | 3.774 | 0.055 | 0.041 |
| Markov Trajectory Entropy | CAP6 to CAP3 | 0.832 | 0.364 | 0.010 | 2.768 | 0.100 | 0.03 |
| Markov Trajectory Entropy | CAP7 to CAP3 | 1.267 | 0.264 | 0.015 | 3.457 | 0.066 | 0.037 |

**Note:** This table presents features that showed significant group differences (*p* < 0.05) in basic model. For each feature, the table shows how these differences are affected when controlling for medication (Medication Model) or illness duration (Duration Model). F-value represents the strength of the group effect; p-value shows the statistical significance; Effect Size is presented as partial eta squared. Basic Model controls for age, sex, and education; Medication Model adds CPZ equivalent dose; Duration Model adds illness duration. Significance levels: * *p* < 0.05,  + Significant after FDR correction.

**Table S5. Significant Post-hoc Comparisons of CAP Features Across HC, SCH_Neg, and SCH_Non_Neg Groups in Medication and Duration Models**

| **Feature** | **CAP** | **Group_Comparison** | **Med**  **Mean_A** | **Med**  **Mean_B** | **Med**  **t-value** | **Med**  **p-value** | **Dur**  **Mean_A** | **Dur**  **Mean_B** | **Dur_**  **t-value** | **Dur_**  **p-value** |
| --- | --- | --- | --- | --- | --- | --- | --- | --- | --- | --- |
| iSAR (overall) | CAP2 | HC vs SCH_Neg | - | - | - | - | 0.766 | 0.751 | -2.25 | **0.027*+** |
| iSAR (overall) | CAP2 | SCH_Neg vs SCH_Non_Neg | 0.75 | 0.766 | 2.457 | **0.016*+** | 0.751 | 0.764 | 2.323 | **0.022*+** |
| Occurrence Rate | CAP5 | HC vs SCH_Neg | - | - | - | - | 0.049 | 0.035 | -2.56 | **0.012*+** |
| Transition Probability | CAP5 to CAP6 | HC vs SCH_Neg | - | - | - | - | 0.104 | 0.233 | 2.187 | **0.031*+** |
| Transition Probability | CAP5 to CAP6 | SCH_Neg vs SCH_Non_Neg | - | - | - | - | 0.233 | 0.117 | -2.756 | **0.007**+** |

**Note:** This table presents only significant pairwise comparisons for features with significant main effects. For each comparison, mean values for both groups (A and B), t-value, and p-values are shown for both medication and duration models where applicable. 'Med' indicates Medication Model results (controlling for age, sex, education, and CPZ); 'Dur' indicates Duration Model results (controlling for age, sex, education, and illness duration). Significance levels: * *p* < 0.05, ** *p* < 0.01, *** *p* < 0.001. + indicates significance after FDR correction. '-' indicates comparisons where the model did not yield significant results or where data was not available for that model.

**Table S6. Percentage (%) of Brain Regions with Positive and Negative Activation in Each CAP State**

| **CAP** | **VN+** | **VN-** | **SMN+** | **SMN-** | **DAN+** | **DAN-** | **SAN+** | **SAN-** | **LN+** | **LN-** | **CN+** | **CN-** | **DMN+** | **DMN-** | **SCN+** | **SCN-** |
| --- | --- | --- | --- | --- | --- | --- | --- | --- | --- | --- | --- | --- | --- | --- | --- | --- |
| 1 | 37.75 | 0 | 28.48 | 2.9 | 1.32 | 19.57 | 0 | 27.54 | 6.62 | 2.9 | 0 | 34.78 | 25.83 | 9.42 | 0 | 2.9 |
| 2 | 28.21 | 1.34 | 48.72 | 0 | 8.33 | 9.4 | 11.54 | 8.72 | 0 | 4.03 | 0.64 | 32.89 | 2.56 | 41.61 | 0 | 2.01 |
| 3 | 0 | 16.88 | 48.61 | 0 | 15.97 | 3.25 | 25 | 1.3 | 0 | 9.74 | 7.64 | 17.53 | 0 | 51.3 | 2.78 | 0 |
| 4 | 1.35 | 26.42 | 0 | 47.8 | 12.84 | 7.55 | 6.08 | 13.21 | 4.05 | 0 | 33.11 | 0.63 | 41.22 | 3.14 | 1.35 | 1.26 |
| 5 | 11.97 | 6.96 | 27.46 | 21.74 | 8.45 | 12.17 | 17.61 | 10.43 | 7.75 | 0.87 | 7.04 | 19.13 | 18.31 | 26.96 | 1.41 | 1.74 |
| 6 | 0 | 38.31 | 6.87 | 24.03 | 14.5 | 1.3 | 30.53 | 0 | 2.29 | 7.14 | 32.06 | 0 | 9.16 | 29.22 | 4.58 | 0 |
| 7 | 8.28 | 0 | 0 | 51.41 | 4.83 | 14.08 | 2.07 | 24.65 | 9.66 | 0 | 20.69 | 6.34 | 54.48 | 0.7 | 0 | 2.82 |
| 8 | 7.89 | 16.45 | 20.18 | 26.32 | 18.42 | 6.58 | 9.65 | 15.13 | 0.88 | 7.24 | 22.81 | 4.61 | 18.42 | 22.37 | 1.75 | 1.32 |

**Note:** VN = Visual Network; SMN = Somatomotor Network; DAN = Dorsal Attention Network; SAN = Salience/Ventral Attention Network; LN = Limbic Network; CN = Control Network; DMN = Default Mode Network; SCN = Subcortical Network. "+" indicates positive activation

# Reference

An, Z., Tang, K., Xie, Y., Tong, C., Liu, J., Tao, Q., Yan, C.-G., Chen, X., Cao, L.-P., Chen, W., Cheng, Y.-Q., Fang, Y.-R., Gong, Q.-Y., Guo, W.-B., Kuang, L., Li, B.-J., Li, T., Liu, Y.-S., Liu, Z.-N., . . . Feng, Y. (2024). Aberrant resting-state co-activation network dynamics in major depressive disorder. *Translational Psychiatry*, *14*(1), 1. <https://doi.org/10.1038/s41398-023-02722-w>

Caliński, T., & and Harabasz, J. (1974). A dendrite method for cluster analysis. *Communications in Statistics*, *3*(1), 1-27. <https://doi.org/10.1080/03610927408827101>

Davies, D. L., & Bouldin, D. W. (1979). A Cluster Separation Measure. *IEEE Transactions on Pattern Analysis and Machine Intelligence*, *PAMI-1*(2), 224-227. <https://doi.org/10.1109/TPAMI.1979.4766909>

Ekroot, L., & Cover, T. M. (1993). The entropy of Markov trajectories. *IEEE Transactions on Information Theory*, *39*(4), 1418-1421.

Janes, A. C., Peechatka, A. L., Frederick, B. B., & Kaiser, R. H. (2020). Dynamic functioning of transient resting-state coactivation networks in the Human Connectome Project. *Hum Brain Mapp*, *41*(2), 373-387. <https://doi.org/10.1002/hbm.24808>

Rousseeuw, P. J. (1987). Silhouettes: A graphical aid to the interpretation and validation of cluster analysis. *Journal of Computational and Applied Mathematics*, *20*, 53-65. <https://doi.org/https://doi.org/10.1016/0377-0427(87)90125-7>

Sun, F., Cui, D., Jiao, Q., Niu, J., Zhang, X., Shi, Y., Liu, H., Ouyang, Z., Yu, G., Dou, R., Guo, Y., Dong, L., & Cao, W. (2024). The co-activation pattern between the DMN and other brain networks affects the cognition of older adults: evidence from naturalistic stimulation fMRI data. *Cereb Cortex*, *34*(1). <https://doi.org/10.1093/cercor/bhad466>

Yang, H., Zhang, H., Di, X., Wang, S., Meng, C., Tian, L., & Biswal, B. (2021). Reproducible coactivation patterns of functional brain networks reveal the aberrant dynamic state transition in schizophrenia. *Neuroimage*, *237*, 118193. <https://doi.org/10.1016/j.neuroimage.2021.118193>

Zhang, Y., Lin, L., Zhou, D., Song, Y., Stein, A., Zhou, S., Xu, H., Zhao, W., Cong, F., Sun, J., Li, H., & Du, F. (2024). Age-related unstable transient states and imbalanced activation proportion of brain networks in people with autism spectrum disorder: a resting-state fMRI study using co-activation pattern analyses. *Network Neuroscience*, 1-43. <https://doi.org/10.1162/netn_a_00396>
